# HIF inhibitor 32-134D eradicates murine hepatocellular carcinoma in combination with anti-PD1 therapy

**DOI:** 10.1172/JCI156774

**Published:** 2022-05-02

**Authors:** Shaima Salman, David J. Meyers, Elizabeth E. Wicks, Sophia N. Lee, Emmanuel Datan, Aline M. Thomas, Nicole M. Anders, Yousang Hwang, Yajing Lyu, Yongkang Yang, Walter Jackson, Dominic Dordai, Michelle A. Rudek, Gregg L. Semenza

**Affiliations:** 1 Armstrong Oxygen Biology Research Center,; 2 Institute for Cell Engineering,; 3 McKusick-Nathans Department of Genetic Medicine,; 4 Department of Pharmacology and Molecular Sciences,; 5 Department of Radiology and Radiological Science,; 6 Department of Oncology and Sidney Kimmel Comprehensive Cancer Center,; 7 Division of Clinical Pharmacology, Johns Hopkins University School of Medicine, Baltimore, Maryland, USA.

**Keywords:** Oncology, Therapeutics, Cancer immunotherapy, Liver cancer, Transcription

## Abstract

Hepatocellular carcinoma (HCC) is a major cause of cancer mortality worldwide and available therapies, including immunotherapies, are ineffective for many patients. HCC is characterized by intratumoral hypoxia, and increased expression of hypoxia-inducible factor 1**α** (HIF-1**α**) in diagnostic biopsies is associated with patient mortality. Here we report the development of 32-134D, a low-molecular-weight compound that effectively inhibits gene expression mediated by HIF-1 and HIF-2 in HCC cells, and blocks human and mouse HCC tumor growth. In immunocompetent mice bearing Hepa1-6 HCC tumors, addition of 32-134D to anti-PD1 therapy increased the rate of tumor eradication from 25% to 67%. Treated mice showed no changes in appearance, behavior, body weight, hemoglobin, or hematocrit. Compound 32-134D altered the expression of a large battery of genes encoding proteins that mediate angiogenesis, glycolytic metabolism, and responses to innate and adaptive immunity. This altered gene expression led to significant changes in the tumor immune microenvironment, including a decreased percentage of tumor-associated macrophages and myeloid-derived suppressor cells, which mediate immune evasion, and an increased percentage of CD8^+^ T cells and natural killer cells, which mediate antitumor immunity. Taken together, these preclinical findings suggest that combining 32-134D with immune checkpoint blockade may represent a breakthrough therapy for HCC.

## Introduction

Hepatocellular carcinoma (HCC) is the third leading cause of cancer mortality worldwide and incidence in the United States has tripled over the last 2 decades (1–3). At the time of diagnosis, more than two-thirds of HCC patients have advanced disease, which is often intractable to available therapies and has a 5-year survival of less than 15% (3). The first approved drug for advanced HCC, which was the tyrosine kinase inhibitor (TKI) sorafenib, provided only a modest survival benefit of 2 to 3 months with low response rates, high toxicity often requiring dose reduction or treatment interruption, and frequent development of resistance, and was followed by several other TKIs (lenvatinib, regorafenib, cabozantinib) and ramucirumab, an antibody against vascular endothelial growth factor A (VEGFA) receptor 2 (4–6).

Immune checkpoint blockade with antibodies that target programmed death 1 (PD1) or PD1 ligand 1 (PDL1) has revolutionized the treatment of advanced melanoma, non–small cell lung cancer, and renal cell carcinoma (RCC) (7–9). PDL1, which is expressed on tumor and stromal cells, binds to PD1 on T cells and triggers exhaustion or apoptosis. Nivolumab, an anti-PD1 antibody, was granted FDA approval for HCC based on phase II trial data, but a phase III trial versus sorafenib as first-line therapy did not meet its primary endpoint with respect to overall survival (OS), and a phase III trial of pembrolizumab, another anti-PD1 antibody, also failed versus placebo as second-line HCC therapy (5, 10). A phase III trial of atezolizumab, an anti-PDL1 antibody, in combination with bevacizumab, an antibody against VEGFA, led to a 2.5-month improvement in progression-free survival as compared with sorafenib, although more than half of patients receiving the combination therapy suffered grade 3 or 4 adverse events (11). Most recently, the combination of antibodies against PD1 (nivolumab) and CTLA4 (ipilimumab) was approved for treatment of HCC patients, who previously received sorafenib, with an overall response rate of 33% (12). Many patients may fail to respond to immune checkpoint inhibitors because of the coexistence of other mechanisms of immune evasion, such as the production of adenosine, which binds to its cognate receptor on T cells to trigger apoptosis (13), or the absence of sufficient T and NK cells in the tumor to serve as targets for anti-PD1 (14).

Intratumoral hypoxia is a major driving force for cancer progression (15–19). In breast cancers that are accessible to direct measurement in situ, median *P*O_2_ levels are only 10 mmHg (1.4% O_2_) and increased mortality is associated with a median *P*O_2_ of less than 10 mmHg (19). In liver metastases of colorectal cancer, the median *P*O_2_ was 6 mmHg as compared with 30 mmHg in normal liver tissue (20). Analysis of orthotopic rat HCC tumors revealed that median *P*O_2_ values ranged from 0.2 to 0.8 mmHg, as compared with 45 mmHg in normal rat liver (21). Examination of human HCC patients by dynamic contrast-enhanced magnetic resonance imaging revealed that high intratumoral blood flow was associated with increased OS (22), which is consistent with an association between intratumoral hypoxia and patient mortality.

Hypoxia-inducible factors (HIFs) play critical roles in cancer progression by activating the transcription of a large battery of genes encoding proteins that play key roles in angiogenesis, glucose metabolism, invasion/metastasis, stem cell specification, protumoral inflammatory responses, and tumor immune evasion (18, 23–27). HIFs consist of an O_2_-regulated HIF-1α, HIF-2α, or HIF-3α subunit and a constitutively expressed HIF-1β subunit (28). HIF-α subunits are subjected to O_2_-dependent prolyl hydroxylation, which triggers protein degradation, and O_2_-dependent asparaginyl hydroxylation, which blocks coactivator recruitment (28). In multiple studies involving a range of treatment modalities, as well as a meta-analysis, HIF-1α immunohistochemistry of tumor biopsies has revealed increased expression in over 60% of HCC cases and a significant association with decreased disease-free and OS (29–33) as well as increased risk of recurrence after radiation therapy (34) or surgery (35).

The connection between immune evasion and intratumoral hypoxia was established by groundbreaking studies demonstrating that hypoxia induces increased expression of CD73, the enzyme that mediates production of extracellular adenosine, which binds to adenosine 2A receptors on T lymphocytes and natural killer (NK) cells, thereby suppressing antitumor immunity (13, 17, 36). In mouse models, hypoxic zones within tumors were shown to lack T cells (37, 38). The critical role of intratumoral hypoxia as the stimulus for immunosuppression was also demonstrated by exposing tumor-bearing mice to an ambient O_2_ concentration of 60%, which was sufficient to alter the tumor microenvironment from immunosuppression to antitumor immunity (37). In contrast to the studies described above, which implicated HIF-dependent adenosine production by cancer cells as a non-cell-autonomous mechanism of immunosuppression, other studies supported the hypothesis that HIF activity was required in a cell-autonomous manner for the antitumor activity of CD8^+^ T cells (39, 40). Thus, it was not clear from these prior studies whether the net effect of a systemic HIF inhibitor would be to augment or overcome the observed suppression of antitumor immunity.

We previously screened a library of 3,140 drugs and identified acriflavine as a drug that inhibits HIF activity by disrupting subunit dimerization, and blocks HCC tumor growth and vascularization in mouse models (41). Acriflavine treatment of tumor-bearing mice was recently reported to increase intratumoral CD8^+^ T cells and NK cells (42, 43). However, acriflavine is not an optimal candidate for clinical trials due to its propensity to cause DNA damage and the need to administer HIF inhibitors on a daily long-term basis. Recently, compounds designated PT2385 and PT2977 were shown to selectively block dimerization of HIF-2α with HIF-1β (44) with encouraging clinical trial results (45–47), and PT2977/belzutifan was recently approved by the FDA for the treatment of RCC in patients with von Hippel-Lindau syndrome (47, 48). Although many compounds have been shown to inhibit HIF activity in cultured cells (28), there are no drugs currently approved that target both HIF-1 and HIF-2 for inhibition. In the present study, we interrogated the National Cancer Institute panel of 60 cancer cell lines (NCI-60) for chemical compounds that induced gene expression profiles similar to acriflavine. Based on a lead compound from this screen, we developed HIF inhibitors that are chemically unrelated to acriflavine. Compound 32-134D blocked human HCC tumor xenograft growth and, in combination with anti-PD1 therapy, eradicated mouse HCC tumors by overcoming HIF-dependent suppression of antitumor immunity.

## Results

### Identification of a class of HIF inhibitors.

**Figure 1:**
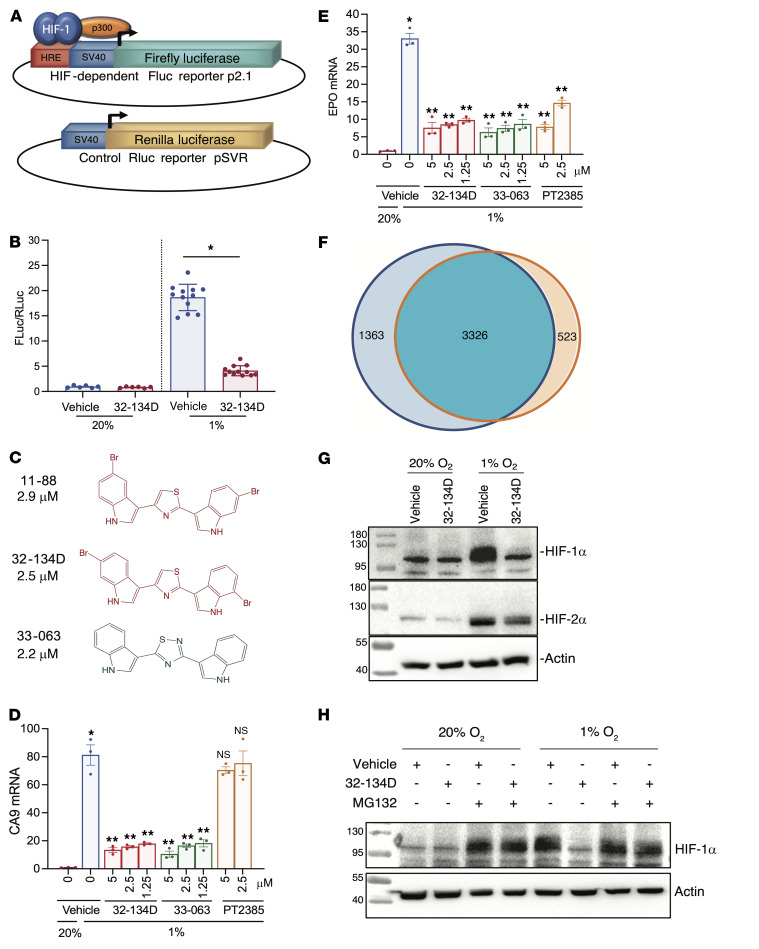
Identification of HIF inhibitors. (**A**) Hep3B-c1 cells were stably transfected with firefly luciferase (FLuc) reporter p2.1, which contains a hypoxia-response element (HRE), and Renilla luciferase (RLuc) reporter pSVR. (**B**) Hep3B-c1 cells were incubated with vehicle (Veh; 0.1% DMSO; blue bars) or 10 μM 32-134D (red bars) at 20% O_2_ (*n =* 6) or 1% O_2_ (*n =* 12) for 24 hours. Cell lysates were assayed for Fluc/Rluc activity (mean ± SEM); *P < 0.05 versus vehicle (χ^2^ test). (**C**) Chemical structures and IC_50_ values for HIF inhibitors. (**D** and **E**) Hep3B cells were exposed to 20% O_2_ with vehicle, or 1% O_2_ with vehicle (blue bar), 32-134D (red bars), 33-063 (green bars), or PT2385 (brown bars) for 24 hours and CA9 (**D**) and EPO (**E**) mRNAs were quantified by RT-qPCR. Data are presented as mean ± SEM (*n =* 3). *P < 0.05 versus 20% O_2_-vehicle; **P < 0.01 versus 1% O_2_-vehicle (ANOVA with Bonferroni’s post hoc test); NS, not significantly different from 1% O_2_-vehicle. (**F**) Hep3B cells were exposed to 20% or 1% O_2_ in the presence of vehicle or 1% O_2_ in the presence of 5 μM 32-134D (*n =* 3 each) for 24 hours. RNA sequencing identified genes with hypoxia-induced expression (blue circle) and genes that were inhibited by 32-134D (orange circle), based on FDR < 0.05 and fold change > 1.5. (**G**) Hep3B cells were exposed to 20% or 1% O_2_ for 24 hours in the presence of vehicle or 5 μM 32-134D, nuclear extracts were prepared, and immunoblot assays were performed. (**H**) Hep3B cells were exposed to 20% or 1% O_2_ for 24 hours with vehicle, 5 μM 32-134D, or 5 μM MG132 (during last 8 hours of exposure), nuclear extracts were prepared, and immunoblot assays were performed.

The NCI CellMiner database contains expression data for over 25,000 mRNAs in 60 human cancer cell lines that have been exposed to more than 20,000 chemical compounds (49). We searched CellMiner for a compound that induced changes in gene expression that were highly correlated with those induced by acriflavine, but which was structurally unrelated to acriflavine. Compounds that satisfied the above criteria were identified and subsequently analyzed in Hep3B-c1 cells (50), which are stably transfected with HIF-dependent reporter plasmid p2.1, in which firefly luciferase (FLuc) coding sequences are located downstream from a hypoxia-response element (HRE) and a basal SV40 promoter; and control reporter pSVR, in which Renilla luciferase (RLuc) coding sequences are downstream of the basal SV40 promoter only (Figure 1A). The FLuc/RLuc ratio in cells exposed to 1% O_2_ for 24 hours is a specific measure of HIF-dependent gene expression. NSC-705870 (hereafter designated 11-88), a bis-bromoindole thiazole compound that had a Pearson’s correlation of 0.475 with acriflavine in the CellMiner database (*P* = 1.2 × 10^–4^), significantly inhibited the FLuc/RLuc ratio in hypoxic Hep3B cells (*P* < 0.05; Figure 1B), with an IC_50_ of 2.9 μM (Figure 1C).

Based on this hit, we synthesized analogs in which either halogen substitutions to the indole groups were altered or the central thiazole was replaced by imidazole, isoxazole, oxadiazole, oxazole, pyrazinone, pyridazine, pyrazine, pyrazole, pyridine, thiadiazole, triazine, or triazole. Among 252 analogs, we identified 27 compounds that inhibited HIF transcriptional activity with IC_50_ less than 3.3 μM in the reporter assay (Supplemental Figure 1; supplemental material available online with this article; https://doi.org/10.1172/JCI156774DS1). We focused on analyzing the effect of the bis-bromoindole thiazole 32-134D and the bis-indole thiadiazole 33-063 on endogenous HIF target gene expression in Hep3B cells. Reverse transcription and quantitative real-time PCR (RT-qPCR) revealed that both of these compounds significantly inhibited (*P* < 0.01) hypoxia-induced expression of the following HIF target genes: *CA9*, which encodes carbonic anhydrase 9 and in Hep3B cells is activated by HIF-1 only (Figure 1D); *ANGPTL4* and *VEGFA*, which encode angiopoietin-like 4 and VEGFA, and are regulated by both HIF-1 and HIF-2 (Supplemental Figure 2, A and B); and *NDRG1* (Supplemental Figure 2C) and *EPO* (Figure 1E), which encode N-myc downstream regulated 1 and erythropoietin, and are activated only by HIF-2 in Hep3B cells. The data indicate that 32-134D and 33-063 inhibit transcription mediated by both HIF-1 and HIF-2. For comparison, we tested the effect of PT2385, which inhibited *EPO*, *NDRG1*, *ANGPTL4*, and *VEGFA* expression but had no effect on *CA9*, which is consistent with its HIF-2–selective mechanism of action (44). None of the compounds had any effect on *RPL13A* expression, which is neither induced by hypoxia nor regulated by HIFs (Supplemental Figure 2D).

RNA sequencing (RNA-seq) was performed on 3 biological replicates of Hep3B cells exposed for 24 hours to vehicle at 20% O_2_, vehicle at 1% O_2_, or 5 μM 32-134D at 1% O_2_. Principal component analysis (PCA) revealed that mRNA expression in the cells exposed to 32-134D at 1% O_2_ was more similar to cells exposed to vehicle at 20% O_2_ as compared with cells exposed to vehicle at 1% O_2_ (Supplemental Figure 2E). There were 3,849 mRNAs with significantly increased expression (FDR < 0.05) of at least 1.5-fold in vehicle-treated Hep3B cells exposed to 1% O_2_ (as compared with 20% O_2_) and hypoxia-induced expression of 3,326 genes (86%) was inhibited by 32-134D (Figure 1F). Overall, 32-134D inhibited the expression of 4,689 genes, of which the 3,326 hypoxia-induced genes represented 71% of the total. Kaplan-Meier analysis of 364 HCC patients, using intratumoral expression of a HIF signature consisting of 15 genes (Supplemental Table 1) with expression that was induced by hypoxia and inhibited by 32-134D, revealed that HIF expression in the primary tumor was associated with decreased patient survival from 82 months in patients with intratumoral HIF signature less than the median value to 42 months in patients with HIF signature greater than the median (*P* = 0.006; Supplemental Figure 2F). Taken together, these results indicate that the HIF transcriptome is the major target of 32-134D in hypoxic Hep3B cells and that increased intratumoral expression of HIF target genes is associated with HCC patient mortality.

Immunoblot assays revealed that treatment of Hep3B cells with 32-134D for 24 hours inhibited the expression of HIF-1α and, to a lesser extent, HIF-2α protein expression (Figure 1G), with no effect on HIF-1α or HIF-2α mRNA expression (Supplemental Figure 2, G and H). Coadministration of the proteasome inhibitor MG-132 blocked the effect of 32-134D on HIF-1α protein levels (Figure 1H). Chromatin immunoprecipitation (ChIP)-qPCR assays revealed that 32-134D treatment impaired the hypoxia-induced recruitment of HIF-1 (HIF-1α and HIF-1β), HIF-2 (HIF-2α and HIF-1β), and coactivator p300 to HREs of the *CA9*, *EPO*, and *ANGPTL4* genes (Supplemental Figure 3). In contrast, PT2385 inhibited the hypoxia-induced recruitment of HIF-2 and p300 to the *ANGPTL4* and *EPO* HREs, not to the *CA9* HRE, which is occupied by HIF-1 only. Taken together, the results presented in Figure 1 and Supplemental Figures 1–3 indicate that 32-134D induces HIF-α subunit degradation, thereby inhibiting activation of HIF-1 and HIF-2 target gene transcription.

### Effects of 32-134D on human HCC tumor xenografts.

**Figure 2:**
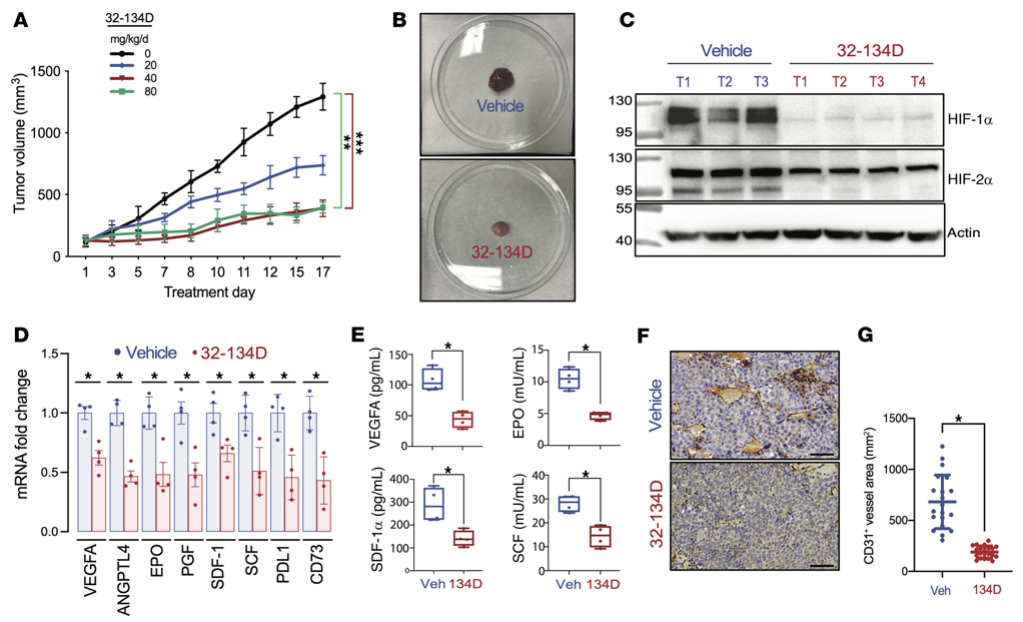
Effect of 32-134D on Hep3B tumor xenograft growth and vascularization. (**A**) Female nude mice received a subcutaneous injection of 5 × 10^6^ Hep3B cells. When tumors reached a volume of 150 mm^3^ (designated treatment day 1), the mice were randomized to receive a daily intraperitoneal injection of 32-134D at a dose of 0 (blue), 20 (black), 40 (red), or 80 (green) mg/kg. Data are presented as mean tumor volume (± SEM; *n =* 4 each). **P < 0.01, ***P < 0.001 (ANOVA with Bonferroni’s post hoc test). (**B**) Gross pathology of tumors harvested from vehicle-treated (top panel) and 32-134D–treated (bottom panel) mice. (**C**) Nuclear extracts prepared from tumors were assayed by immunoblotting using antibodies against the indicated proteins. (**D**) Total RNA was isolated from tumor tissue and analyzed by RT-qPCR using primers specific for the indicated mRNAs and results (mean ± SEM, *n =* 4) were normalized to the mean value for tumors from vehicle-treated mice. (**E**) ELISA for the indicated proteins was performed using aliquots of tumor lysates (mean ± SEM; *n =* 3–4 tumors each). *P < 0.05 (Mann-Whitney test). (**F**) Formalin-fixed and paraffin-embedded tumor sections were analyzed by immunohistochemistry using an antibody against CD31 to identify vascular endothelial cells. Scale bar: 100 μm. The total CD31^+^ vessel area per field was quantified using ImageJ (mean ± SEM; *n =* 4 tumors with 5 sections per tumor). *P < 0.05 (Student’s *t* test).

Having demonstrated the effect of 32-134D on HIF-dependent gene expression in cultured Hep3B cells, we next sought to analyze the effect of HIF inhibitor 32-134D on tumor xenograft growth and vascularization. Hep3B cells were injected subcutaneously into nude (*nu/nu*) mice and when tumors reached a volume of 150 mm^3^ (designated day 1), the mice were treated with vehicle or 32-134D by daily intraperitoneal injection. Compared with vehicle treated mice, partial growth inhibition was observed at 20 mg/kg (*P* < 0.01) and maximal growth inhibition was observed at 40 and 80 mg/kg (*P* < 0.001) (Figure 2A and Supplemental Figure 4A). Treatment with 32-134D for 17 days had no effect on mouse appearance, behavior, or body weight (Supplemental Figure 4B). We treated additional mice with 32-134D at a dose of 40 mg/kg versus vehicle and confirmed inhibition of tumor growth without effects on body weight (Supplemental Figure 5, A–C). The tumors harvested from 32-134D–treated mice were of significantly decreased mass (*P* < 0.05; Supplemental Figure 5D) and demonstrated pallor in comparison to the bloody appearance of tumors from vehicle-treated mice (Figure 2B), suggesting effects on tumor vascularization.

Immunoblot assays of tumor lysates revealed that 32-134D resulted in an almost complete loss of HIF-1α and HIF-2α protein expression, with no effect on actin levels (Figure 2C). Intratumoral HIF-1β protein levels were not affected by treatment with 32-134D (Supplemental Figure 5E). Analysis of tumor RNA revealed a significant decrease in the expression of mRNAs encoding (a) angiogenic growth factors, including ANGPTL4, EPO, and placental growth factor (PGF); (b) proteins mediating immune evasion, including CD73 and PDL1; and (c) proteins with effects on both angiogenesis and immunity, including VEGFA, stroma-derived factor 1 (SDF-1; also known as CXCL12), and stem cell factor (SCF; also known as KIT ligand [KITLG]) in response to 32-134D treatment (*P* < 0.05; Figure 2D). Tumor lysates were subjected to ELISA, which revealed significantly decreased expression of VEGFA, EPO, SDF-1α, and SCF protein in tumor lysates from mice treated with 32-134D (*P* < 0.05; Figure 2E). Immunohistochemistry using an antibody against CD31, which is expressed by vascular endothelial cells, demonstrated significantly decreased blood vessel area in tumors from 32-134D–treated mice (*P* < 0.05; Figure 2, F and G, and Supplemental Figure 6). Taken together, the data presented in Figure 2 and Supplemental Figures 4–6 demonstrate that treatment with 32-134D inhibits human HCC tumor xenograft growth and angiogenesis by inhibiting the expression of multiple HIF target genes.

### Effects of 32-134D on Hepa1-6 mouse HCC cells and tumors.

**Figure 3:**
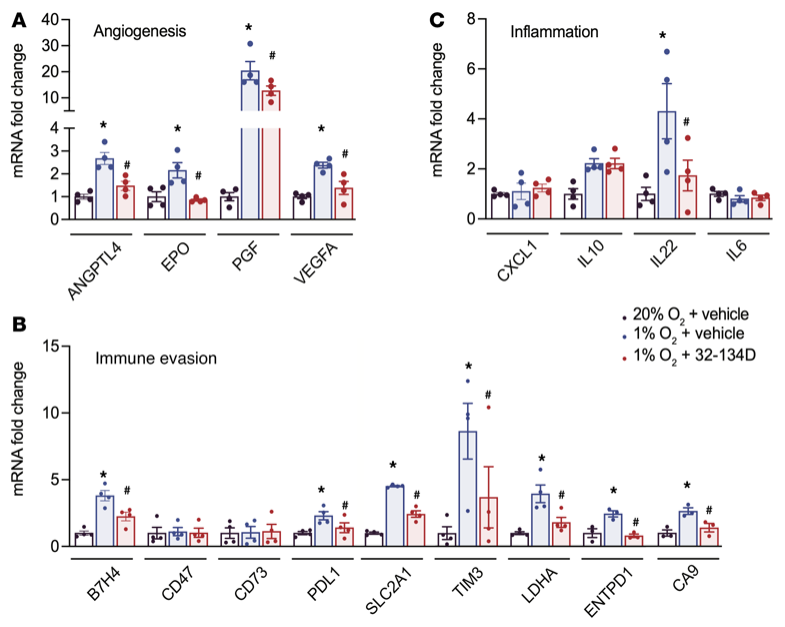
Effect of 32-134D treatment on hypoxia-induced gene expression in Hepa1-6 cells. (**A**–**C**) Cells were exposed to 20% O_2_ and vehicle (white bars), 1% O_2_ and vehicle (blue bars), or 1% O_2_ and 32-134D (red bars) for 24 hours and mRNAs were quantified by RT-qPCR and normalized to white (mean ± SEM, *n =* 4). *P < 0.05 versus white; ^#^*P* < 0.05 versus blue (ANOVA with Bonferroni’s post hoc test).

Next, we analyzed Hepa1-6, which is a mouse HCC cell line. Treatment of hypoxic Hepa1-6 mouse HCC cells with 32-134D or 33-063 revealed significant dose-dependent inhibition of hypoxia-induced ANGPTL4, EPO, PGF, and VEGFA mRNA expression with no effect on RPL13A (*P* < 0.05; Figure 3A and Supplemental Figure 7). PT2385 was a less potent inhibitor of ANGPTL4, PGF, and VEGFA mRNA expression (Supplemental Figure 7). Analysis of genes that are induced by HIFs in breast cancer and promote immune evasion (42) revealed that CD47 and CD73 were not induced in hypoxic Hepa1-6 cells, whereas PDL1 mRNA was induced in vehicle-treated but not in 32-134D–treated cells under hypoxic conditions (Figure 3B). B7H4 and TIM3 mRNA, which are encoded by the *Vtcn1* and *Havcr2* genes, respectively, and like PDL1, encode immune checkpoint receptors that are HIF regulated (51, 52) and associated with patient mortality in HCC (53), showed hypoxia-induced expression in vehicle-treated but not in 32-134D–treated cells (*P* < 0.05; Figure 3B). Treatment with 32-134D also inhibited hypoxia-induced expression of SLC2A1, LDHA, ENTPD1, and CA9 mRNA, which play roles in both tumor metabolism and immune evasion (*P* < 0.05; Figure 3B and Supplemental Table 4). CXCL1, IL-6, and IL-10 were not induced by hypoxia, whereas hypoxia-induced IL-22 expression was inhibited by 32-134D (*P* < 0.05; Figure 3C).

**Figure 4:**
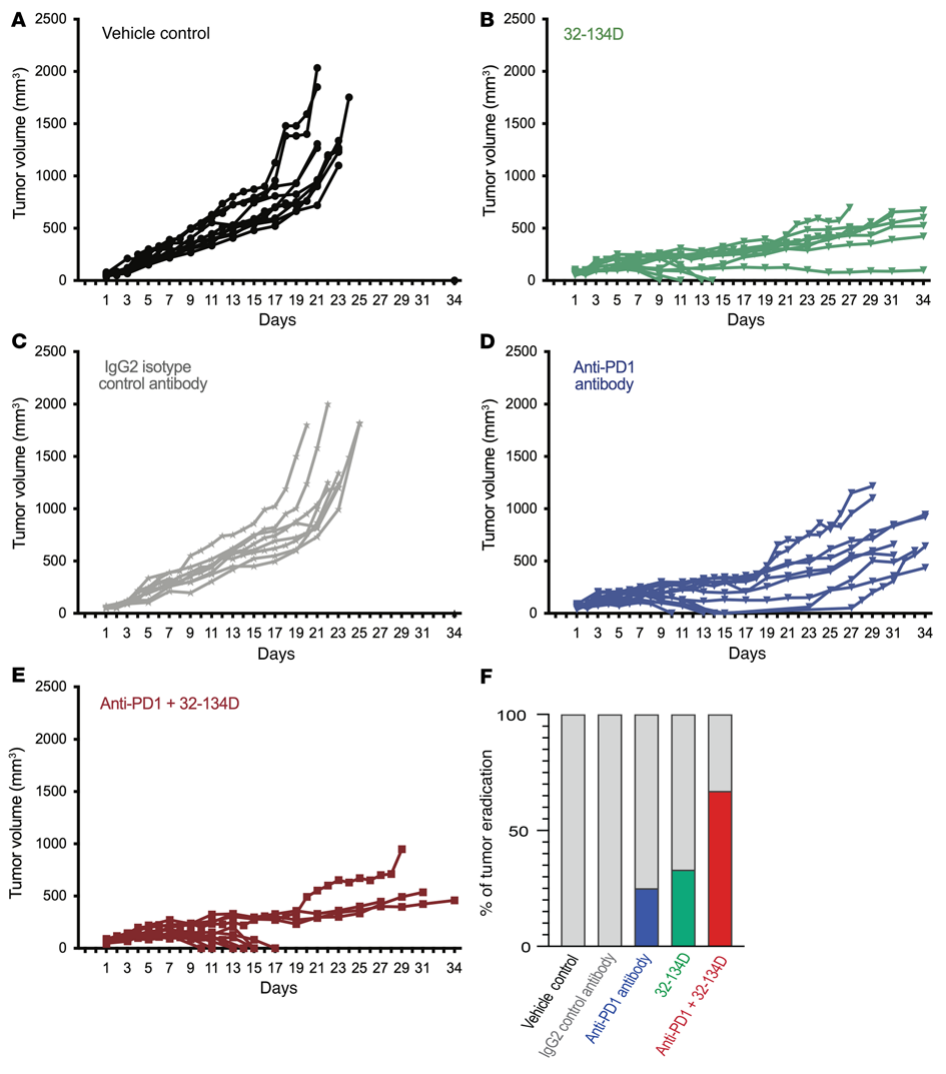
Effect of anti-PD1 and 32-134D on Hepa1-6 tumor growth in syngeneic mice. C57L mice were injected with Hepa1-6 HCC cells subcutaneously and when tumors became palpable, they were randomized to receive intraperitoneal injection of vehicle (**A**) or 32-134D (40 mg/kg; **B**) daily; IgG_2a_ isotype control (**C**) or anti-PD1 (**D**) antibody every 3 days; or both anti-PD1 and 32-134D (**E**). The percentage of mice in each treatment group with tumor eradication on day 34 is shown (**F**; blue, green, and red bars).

To test whether 32-134D inhibits HCC growth in an immunocompetent mouse model, we injected Hepa1-6 HCC cells into the flank of syngeneic C57L mice (54). When tumors became palpable (treatment day 1), mice received intraperitoneal injections of 32-134D (40 mg/kg/day) versus vehicle control, or anti-PD1 antibody versus IgG2a isotype control (200 μg on days 1, 4, 7, 10, 13, and 16), or both 32-134D and anti-PD1. Tumors grew very rapidly in all mice treated with vehicle or IgG2a, necessitating euthanasia on or before day 24 (Figure 4, A and C). Tumors regressed in 5 out of 12 mice treated with anti-PD1, but 2 tumors recurred after the last antibody treatment (on day 16) for a tumor eradication rate of only 25% (Figure 4D). Treatment with 32-134D resulted in inhibition of tumor growth in all mice and tumor eradication was achieved in 4 of 12 mice (Figure 4B). Among mice treated with the combination of 32-134D and anti-PD1, tumor growth was decreased and tumor eradication occurred in 8 of 12 mice, with no recurrences after antibody treatment was discontinued (Figure 4E). Thus, addition of 32-134D increased the percentage of mice with a complete response to anti-PD1 immune checkpoint blockade from 25% to 67% (Figure 4F).

**Figure 5:**
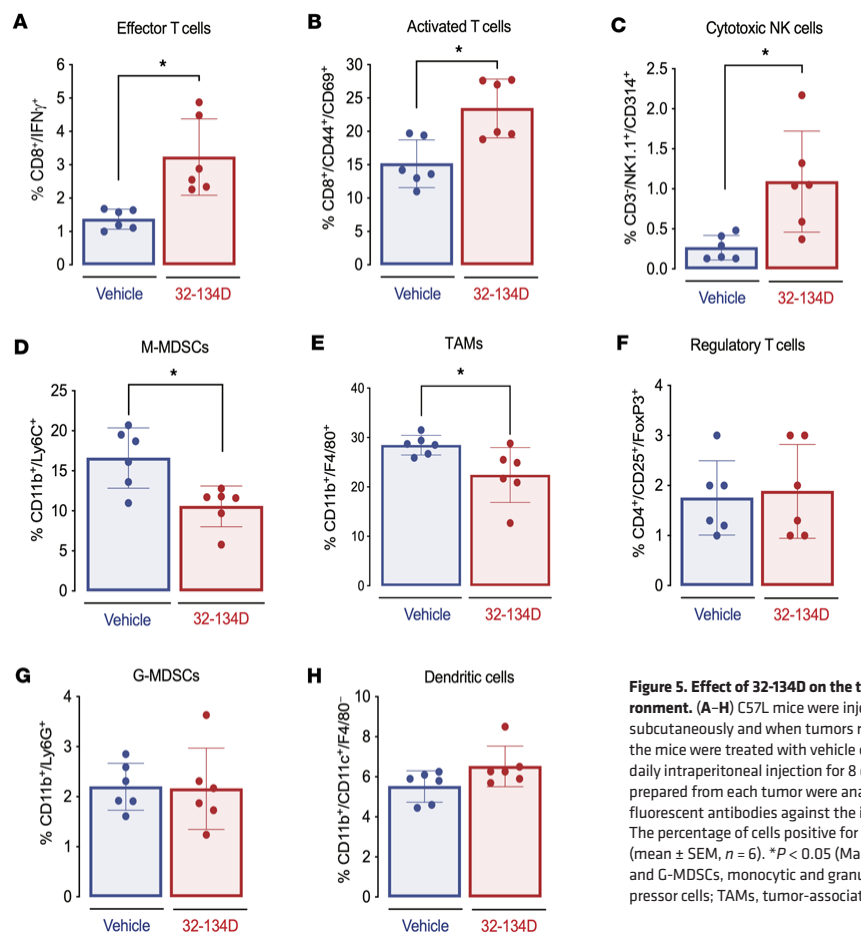
Effect of 32-134D on the tumor immune microenvironment. (**A**–**H**) C57L mice were injected with Hepa1-6 HCC cells subcutaneously and when tumors reached a volume of 200 mm^3^, the mice were treated with vehicle or 32-134D (40 mg/kg) by daily intraperitoneal injection for 8 days. Single-cell suspensions prepared from each tumor were analyzed by flow cytometry using fluorescent antibodies against the indicated cell surface proteins. The percentage of cells positive for the indicated markers is shown (mean ± SEM, *n =* 6). *P < 0.05 (Mann-Whitney test). M-MDSCs and G-MDSCs, monocytic and granulocytic myeloid-derived suppressor cells; TAMs, tumor-associated macrophages.

To analyze the mechanism by which 32-134D increased the response to anti-PD1 therapy, Hepa1-6 tumor–bearing mice were treated with 32-134D or vehicle, and immune cell populations in well-established 200-mm^3^ tumors were analyzed by flow cytometry (Supplemental Figure 8). Within 8 days, HIF inhibitor therapy significantly increased the percentage of CD8^+^IFN-γ^+^ effector T cells, CD8^+^CD44^+^CD69^+^ activated T cells, and NK1.1^+^CD3^–^CD314^+^ activated NK cells, which are critical effectors of antitumor immunity and targets of anti-PD1 therapy (*P* < 0.05; Figure 5, A–C). The pivotal role of CD8^+^ T cells and NK cells in antitumor immunity is underscored by the fact that expression of CD8A and CD8B mRNA as well as KLRK1 and KLRB1 mRNA (encoding the human homologs of CD314 and NK1.1, respectively) in HCC is associated with patient survival (*P* < 0.05; Supplemental Table 2). By contrast, 32-134D treatment led to a decreased percentage of intratumoral CD11b^+^F4/80^+^ tumor-associated macrophages (TAMs) and CD11b^+^Ly6C^+^ monocytic myeloid-derived suppressor cells (M-MDSCs) (*P* < 0.05; Figure 5, D and E), which are 2 immune cell populations that are critical mediators of immunosuppression in HCC (55). Treatment with 32-134D resulted in a 3-fold increase in the ratio of effector T cells to TAMs. The percentage of regulatory T cells, granulocytic MDSCs (G-MDSCs), and dendritic cells within Hepa1-6 tumors was not significantly affected by 32-134D treatment (Figure 5, F–H).

**Figure 6:**
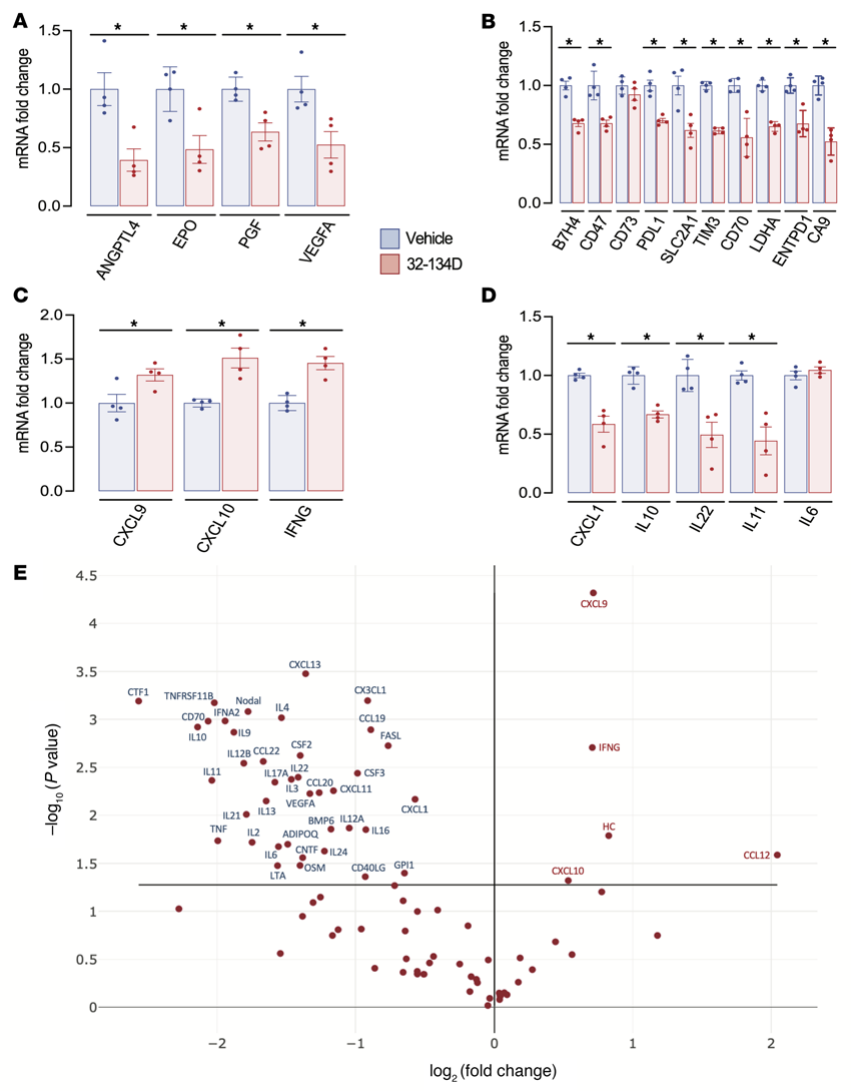
Effect of 32-134D on intratumoral gene expression. (**A**–**D**) C57L mice were injected with Hepa1-6 HCC cells subcutaneously and when tumors reached a volume of 200 mm^3^, the mice were treated with vehicle (blue bars) or 32-134D (40 mg/kg; red bars) by daily intraperitoneal injection for 8 days. The tumors were harvested and mRNA was quantified by RT-qPCR and normalized to blue (mean ± SEM, *n =* 4). *P < 0.05 versus blue (ANOVA with Bonferroni’s post hoc test). (**E**) Effect of 32-134D on intratumoral expression of cytokines and chemokines. Total RNA isolated from tumors of 32-134D–treated versus vehicle-treated mice (*n =* 3 each) was analyzed using an RT-qPCR array and the ratio of mean expression (32-134D/vehicle) was determined. mRNAs with a significant difference between groups (*P* < 0.05, Student’s *t* test) are annotated.

Analysis of mRNA expression in tumor tissue by RT-qPCR revealed that 32-134D treatment significantly decreased the expression of multiple mRNAs encoding angiogenic factors (ANGPTL4, EPO, PGF, and VEGFA) (*P* < 0.05; Figure 6A) and proteins mediating immunosuppression (B7H4/VTCN1, CD47, CD73, PDL1, SLC2A1/GLUT1, HAVCR2/TIM3, CD70, LDHA, ENTPD1/CD39, and CA9) (*P* < 0.05; Figure 6B and Supplemental Table 4), whereas 32-134D treatment increased the expression of CXCL9, CXCL10, and IFN-γ (*P* < 0.05; Figure 6C), which are critical for recruitment and activation of CD8^+^ T cells and NK cells. By contrast, the expression of mRNAs encoding the chemokine CXCL1 and interleukins IL-6 and IL-10, which recruit/activate TAMs and/or MDSCs (Supplemental Table 4), was inhibited by 32-134D treatment (*P* < 0.05; Figure 6D).

**Figure 7:**
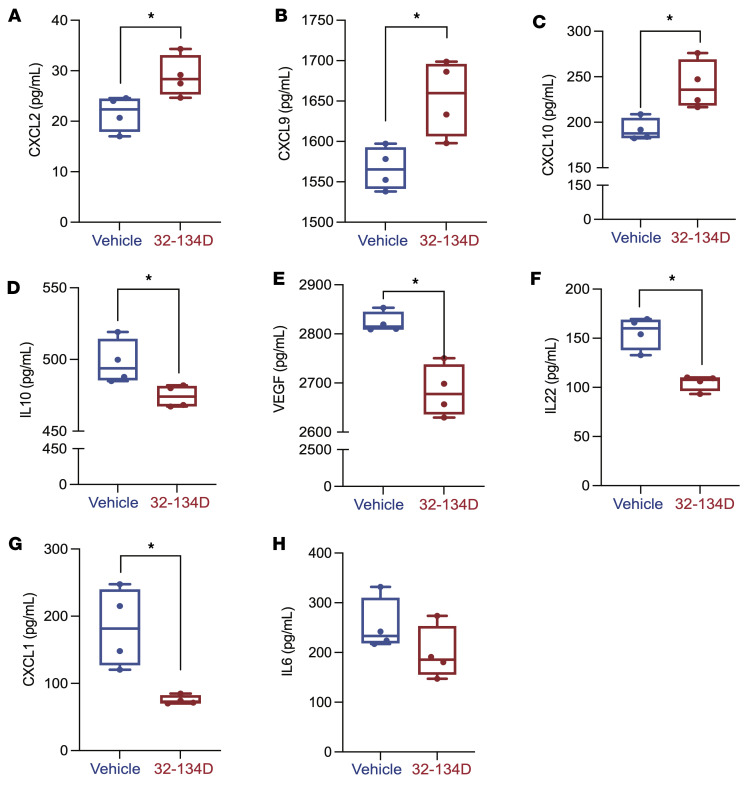
Effect of 32-134D on intratumoral expression of immunoregulatory proteins. (**A**–**H**) Lysates prepared from tumors of 32-134D–treated versus vehicle-treated mice were subjected to ELISA for secreted proteins mediating antitumor immunity (CXCL2, CXCL9, CXCL10) or immunosuppression (CXCL1, IL-6, IL-10, VEGFA). The data are presented as mean ± SEM (*n* = 4 each). *P < 0.05 (Mann-Whitney test).

To extend our characterization of the tumor immune microenvironment, we utilized an RT-qPCR array to analyze mRNAs encoding 84 cytokines, chemokines, interleukins, and other secreted factors, which revealed decreased expression of 40 mRNAs, including those encoding the immunosuppressive cytokines CXCL1, IL-4, IL-6, IL-10, IL-13, and VEGFA (Supplemental Table 4), and increased expression of 5 mRNAs, including CXCL9 and CXCL10, in tumors from 32-134D–treated mice (*P* < 0.05; Figure 6E and Supplemental Table 3). To further extend these findings, protein expression was quantified by performing ELISAs on tumor lysates, which confirmed that tumors from 32-134D–treated mice contained significantly increased levels of CXCL2, CXCL9, and CXCL10 (*P* < 0.05; Figure 7, A–C), which are chemokines that promote antitumor immunity, as well as decreased levels of CXCL1, IL-6, IL-10, and VEGFA (Figure 7, D–H), which are secreted factors that promote establishment of an immunosuppressive tumor microenvironment (Supplemental Table 4).

IL-22 mRNA expression was induced by hypoxia and inhibited by 32-134D in both cultured Hepa1-6 cells (Figure 3C) and Hepa1-6 tumors (Figure 6D). In contrast, decreased CD47, CD70, IL-6, and IL-10 mRNA levels were observed in tumor tissue from 32-134D–treated mice (Figure 6, B and D, and Figure 7) but not in cultured Hepa1-6 cells exposed to 32-134D (Figure 3, B and C), which suggests that 32-134D inhibited the expression of these latter mRNAs in immune or other stromal cell types, where HIFs are known to play critical roles (56–61), rather than in tumor cells. Compound 32-134D inhibited the expression of CNTF, CTF, IL-6, IL-9, IL-11, IL-17A, IL-22, and OSM (*P* < 0.05; Figure 6 and Supplemental Table 3), which are all known to activate JAK/STAT3 signaling, leading to HCC progression (62–68). CXCL1 (69) and B7H4 (70) also play autocrine roles in HCC progression.

### The observed effects of HIF inhibition on gene expression are consistent with therapeutic benefit.

Treatment with 32-134D led to (a) decreased expression of CA9, CXCL1, EPO, LDHA, PGF, SCF/KITLG, and SLC2A1/GLUT1 mRNA, which are all associated with HCC patient mortality; and (b) increased expression of CCL12/CCL2, CXCL2, CXCL9, CXCL10, and HC/C5 mRNA, which are all associated with HCC patient survival (Supplemental Table 2). Taken together, the data presented in Figures 3–7 and Supplemental Figures 7 and 8 demonstrate that treatment of HCC-bearing mice with the HIF inhibitor 32-134D significantly impairs tumor vascularization, alters the tumor immune microenvironment in favor of antitumor immunity, and blocks key metabolic and signal-transduction pathways driving cancer progression, thereby providing a broad molecular and cellular foundation for the increased tumor eradication observed in mice treated with 32-134D, either alone or in combination with anti-PD1 antibody.

### Normal hematologic indices and histology in mice treated with 32-134D.

**Figure 8:**
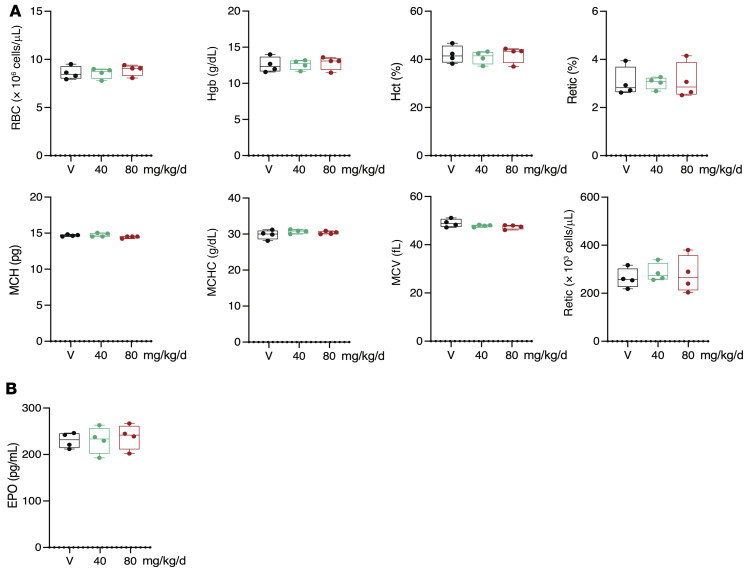
Effect of 32-134D on red blood cell indices. Mice (*n =* 4 per group) were treated with vehicle (V) or 32-134D (40 mg/kg/day) for 14 days and peripheral blood was analyzed for red blood cell count (RBC), hemoglobin (Hgb), hematocrit (Hct), reticulocytes, mean corpuscular hemoglobin (MCH), mean corpuscular hemoglobin concentration (MCHC), mean corpuscular volume (MCV), and absolute reticulocyte count (**A**) or EPO levels in serum (**B**).

To investigate the effect of 32-134D on erythropoiesis, C57BL/6J mice (*n =* 4 per group) received a daily intraperitoneal injection of vehicle or 32-134D at a dose of 40 or 80 mg/kg/day for 14 days. Analysis of peripheral blood samples revealed that red blood cell count, hemoglobin, hematocrit, reticulocyte count, mean corpuscular volume, mouse corpuscular hemoglobin, and mean corpuscular hemoglobin concentration were not significantly different in drug-treated as compared to vehicle-treated mice (Figure 8A). Quantitation of serum EPO levels by ELISA revealed no significant difference between vehicle-treated and 32-134D–treated mice (Figure 8B). Histological analysis of brain, colon, heart, kidney, liver, lungs, and small intestine from mice treated with 32-134D for 14 days revealed no changes compared to vehicle-treated littermates (Supplemental Figure 9).

### Pharmacokinetic analysis of 32-134D.

**Figure 9:**
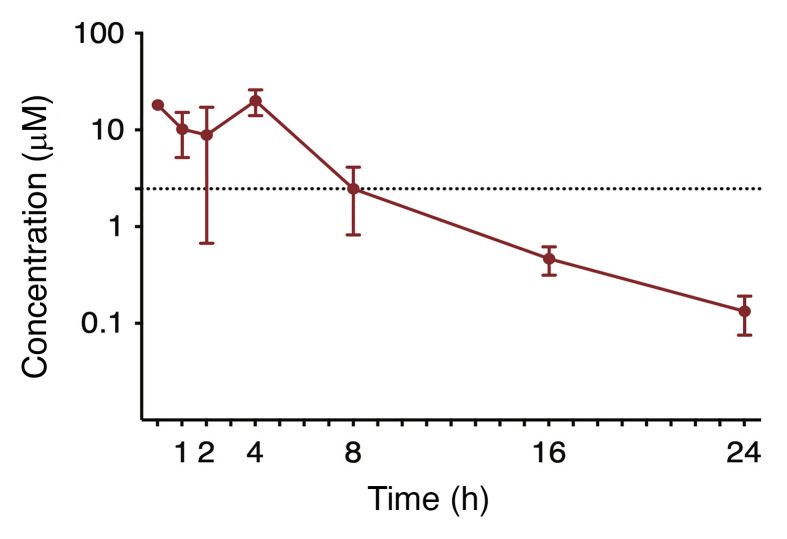
Concentration-time profiles of 32-134D in mice (*n* = 3 per time point) treated with a single dose of 32-134D. Plasma was obtained over 24 hours, with 32-134D concentrations determined by LC-MS/MS. Dashed line represents the in vitro IC_50_ of 32-134D (2.5 μM). Data points and error bars represent mean and SD of 3 replicates, respectively.

We employed liquid chromatography and tandem mass spectrometry (LC-MS/MS) to quantify the plasma concentration of 32-134D over 24 hours following a single intraperitoneal injection of 40 mg/kg (Figure 9). The maximum plasma concentration (C_max_) of 20 μM occurred at 4 hours with a biexponential decline. The plasma concentration of 32-134D exceeded the in vitro IC_50_ of 2.5 μM for at least 8 hours after administration of a single dose. The terminal half-life (*t*_1/2_), apparent clearance (Cl/F), and apparent volume of distribution (V/F) were 4.1 hours, 14.5 mL/min/kg, and 6.1 L/kg, respectively, and the area under the curve extrapolated to infinity (AUC_INF_) was 96.7 μM•h (see Methods for calculation of all parameters). Further studies are required to determine whether steady-state levels are achieved with daily administration.

## Discussion

**Figure 10:**
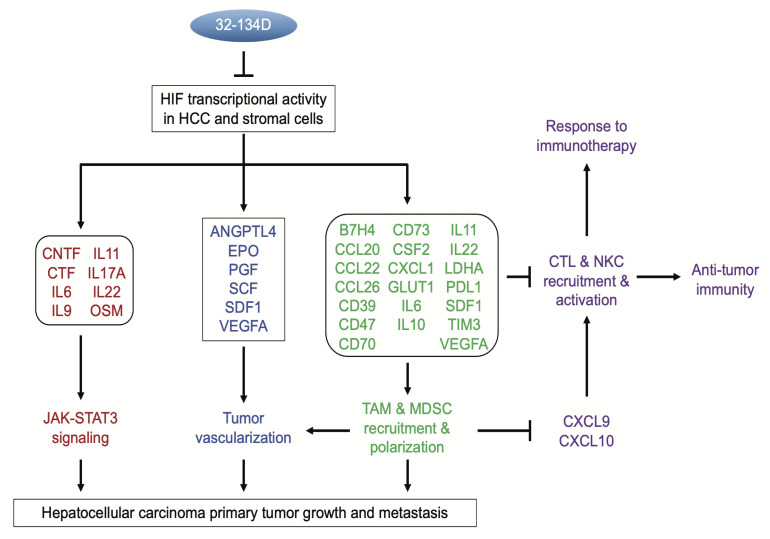
Immunological and other effects of HIF inhibition by 32-134D on HCC progression. The figure summarizes the major HIF target genes that were analyzed in this study. It does not include the many HIF target genes that are involved in other critical aspects of HCC progression that were not analyzed in this study. CTL, cytotoxic T lymphocyte; NKC, natural killer cell.

In this study, we have reported the development of a class of HIF inhibitors that block HIF transcriptional activity in HCC cells by inducing degradation of HIF-1α and HIF-2α protein. Our data demonstrate the profound consequences of inhibiting both HIF-1 and HIF-2 activity in HCC tumors, with effects on tumor growth and vascularization, as well as reprogramming of the tumor immune microenvironment to favor antitumor immunity and improve the response to anti-PD1 therapy (Figure 10). Our results illustrate how the broad effects of HIF inhibition can overcome tumor heterogeneity. We previously showed that in human breast cancer cells HIF-dependent expression of CD47, CD73, and PDL1 inhibited the ability of both the innate and adaptive immune systems to kill cancer cells (42). In breast cancer and melanoma, we demonstrated that HIF-dependent expression of BIRC2 led to decreased CXCL9 expression, which prevented the recruitment of CD8^+^ T cells and NK cells to the tumor (71). By contrast, in cultured Hepa1-6 cells, BIRC2, CD47, and CD73 were not induced by hypoxia. However, in Hepa1-6 tumors, 32-134D inhibited the expression of the checkpoint ligands B7H4 and PDL1, and the checkpoint receptor TIM3, which are all associated with mortality in HCC (53). Treatment with 32-134D led to decreased expression of the Th2 cytokines IL-4 and IL-13, which together with CXCL1, mediate recruitment or maintenance of immunosuppressive MDSCs and TAMs (72, 73). Treatment with 32-134D also decreased the expression of CD70, which has been implicated in immune evasion of cancer cells through induction of T cell exhaustion or apoptosis in glioblastoma (74, 75) and RCC (76).

Cancer cells compete with immune cells for glucose uptake (by SLC2A1/GLUT1), produce lactic acid (by LDHA), and generate an acidic extracellular milieu (through the activity of CA9), all of which are immunosuppressive (77–86). Expression of CXCL9 and CXCL10 was increased in tumors from mice treated with 32-134D, providing a direct mechanism for the increased recruitment of CD8^+^ T cells and NK cells, which increased the response to anti-PD1 therapy. Taken together, these studies have identified multiple mechanisms of immunosuppression that are induced by hypoxia in HCC and that are blocked by treatment with 32-134D. Thus, the net effect of systemic HIF inhibition in HCC is to relieve immunosuppression, as predicted (36), by increasing the recruitment of CD8^+^ T cells and NK cells to the tumor. It should be noted that although the *nu/nu* mice used for the human HCC tumor xenograft experiments are deficient in T and B lymphocytes, they contain NK cells as well as myeloid cells. As a result, it is possible that the antitumor effect of 32-134D in this model was also due at least in part to an unleashing of antitumor immunity.

The cytokine RT-qPCR array also revealed that treatment with the HIF inhibitor led to decreased expression of mRNAs encoding cardiotrophin 1 (CTF1), ciliary neurotrophic factor (CNTF), oncostatin M (OSM), and the interleukins IL-6, IL-9, IL-11, IL-17A, and IL-22. Each of these cytokines, which are produced primarily by immune cells, has been shown to bind to cognate receptors on HCC cells and stimulate JAK/STAT3 signaling, leading to cancer cell proliferation and survival, and to promote cancer stem cell properties that are essential for tumor outgrowth, metastasis, and recurrence (62–68).

Immune checkpoint blockade therapy has had a major impact on cancer by unleashing antitumor immunity that leads to durable tumor regression (87). However, response rates of only 15% to 30% have been observed in most cancer types (88). Combining multiple immune checkpoint blockade therapies is a strategy to increase responders, but among HCC patients, only 33% responded to treatment with anti-CTLA4 and anti-PD1 antibodies (12), which is a regimen that places patients at increased risk for life-threatening autoimmune reactions (89). It is likely that the poor response rate across all human cancers is due in part to the multiplicity of molecular mechanisms (90), many of them regulated by HIFs (25), by which cancer cells evade the immune system. The broad effect of HIF inhibitors provides a means to tamp down the expression of a large battery of genes mediating immune evasion (Figure 10) and thereby improve the therapeutic response to immune checkpoint blockade and other immunotherapies. In particular, the efficacy of many immunotherapies is dependent on the presence of intratumoral CD8^+^ T cells, which are significantly increased by treatment with 32-124D. Finally, the parallel increase in NK cells enables killing of cancer cells that have downregulated the expression of class I MHC proteins to evade killing by T cells (91, 92).

The selective HIF-2 inhibitor belzutifan/PT2977 was recently approved by the US Food and Drug Administration for the treatment of RCC and other tumors in patients with von Hippel-Lindau syndrome (47). The major side effect of the drug is decreased blood hemoglobin levels, which were observed in 90% of patients, with grade 3 anemia present in 7%. A phase I trial of PT2385 reported grade 3 anemia in 10% of patients (46). In mice, 32-134D inhibits intratumoral EPO expression but does not affect serum EPO levels and does not cause anemia over a 2-week treatment course. Based on the favorable safety profile, pharmacokinetics, and efficacy of 32-134D, either alone or in combination with anti-PD1 in mouse models of HCC, further preclinical and clinical development are warranted.

## Methods

### NCI-60 virtual screen.

The Pattern Comparison Analysis Tool and CellMiner Database version 2.1 (Developmental Therapeutics Program, NCI) were accessed at https://discover.nci.nih.gov/cellminer/

### Chemistry.

Detailed methods for the synthesis of 32-134D and 33-063 are presented in the Supplemental Methods.

### Luciferase reporter assay.

Hep3B-c1 cells (50) were seeded on 24-well plates. Cells were treated with compounds the following day and exposed to 20% or 1% O_2_ for 24 hours. FLuc/RLuc ratios were determined using the Dual Luciferase Reporter Assay System (Supplemental Table 6) and the VICTOR Nivo plate reader (PerkinElmer).

### Cell culture.

Human Hep3B and mouse Hepa1-6 cells were purchased from ATCC and grown in high-glucose (4.5 mg/mL) Dulbecco’s modified Eagle’s medium supplemented with 10% (vol/vol) fetal bovine serum and 1% penicillin/streptomycin at 37°C in a 5% CO_2_/95% air incubator (Supplemental Table 6). Human cell line identity was authenticated by analysis of short tandem repeats, and all cell lines were maintained mycoplasma free, using PCR-based assays conducted at the Johns Hopkins University Genetic Resources Core Facility (JHU GRCF). Cells were subjected to hypoxia in a controlled atmosphere chamber (PLAS Labs) with ambient gas mixture containing 1% O_2_ and 5% CO_2_.

### RT-qPCR and RNA-seq.

RNA was extracted using TRIzol Reagent. Quantitative real-time PCR was conducted using SYBR Green PCR Master Mix and reactions were run using a Bio-Rad thermal cycler. Primer sequences and reagent sources are listed in Supplemental Table 5. The mRNA expression of target genes was normalized to the expression of 18S rRNA and the fold change (FC) was calculated based on the threshold cycle (Ct) as FC = 2^–Δ(ΔCt)^, where ΔCt = Ct_target_ _gene_ – Ct_18S__rRNA_ and Δ(ΔCt) = ΔCt_treatment_ – ΔCt_control_. For RNA-seq, total RNA was isolated and treated with DNase (Qiagen). The JHU GRCF High-Throughput Sequencing facility prepared libraries and performed RNA-seq. The RNA-seq data were processed and interpreted using Genialis Expressions software (https://www.genialis.com/expressions/). An automated data analysis pipeline run in the Genialis platform consisted of the following: Sequence quality checks were performed on raw and trimmed reads with FastQC. Bbduk was used to trim adapters and filter out poor quality reads. Trimmed reads were then mapped to the STAR index (ENSEMBL 100) reference genome using the STAR (93) aligner. Gene expression levels were quantified with featureCounts (94), and differential gene expression analyses were performed with DESeq2 (95). Genes exhibiting low expression, i.e., those with an expression count summed over all samples below 10, were filtered out from the differential expression analysis input matrix. Differential expression was determined with an FDR of less than 0.05 and mRNA FC of greater than 1.5 to identify hypoxia-induced genes and genes inhibited by 32-134D. Data used in this study have been deposited in the NCBI Gene Expression Omnibus database (GEO GSE195997).

### Immunoblot assays.

Cells were quickly washed with ice-cold PBS and lysed in 20 mM HEPES (pH 7.6), 1.5 mM MgCl_2_, 10 mM NaCl, 0.2 mM EDTA, 20% glycerol, and 0.1% Triton X-100 supplemented with protease inhibitors (PIs) and briefly centrifuged. Pellets were resuspended in 20 mM HEPES (pH 7.6), 1.5 mM MgCl_2_, 500 mM NaCl, 0.2 mM EDTA, 20% glycerol, 0.1% Triton X-100, and PIs. Blots were probed with antibodies listed in Supplemental Table 6.

### ChIP assays.

Cells were seeded overnight and then exposed to 20% or 1% O_2_ in the presence of compound or vehicle for 16 hours. Protein was cross-linked to DNA by addition of 37% formaldehyde to culture medium for 10 minutes at 37°C and quenched by addition of 0.1 M glycine. Cells were washed with and collected in 5 mL of cold PBS containing PIs. Cells were pelleted and resuspended in 200 μL of SDS lysis buffer (50 mM Tris-HCl [pH 8.1], 10 mM EDTA, 1% SDS) containing PIs and incubated on ice for 10 minutes. Lysates were sonicated to produce DNA fragments ranging from 200 to 900 bp and centrifuged for 10 minutes at 4°C. Supernatants were collected and diluted 10-fold with dilution buffer (16.7 mM Tris-HCl [pH 8.1], 167 mM NaCl, 1.2 mM EDTA, 0.01% SDS, 1.1% Triton X-100) with PIs, precleared with 20 μL of salmon sperm DNA/protein A agarose slurry, and a 20-μL aliquot was reserved as input control. Antibody (2 μg; see Supplemental Table 6) was added and samples were rotated overnight at 4°C. Immune complexes were precipitated with 50 μL of salmon sperm DNA/protein A agarose slurry. Pelleted beads were washed serially using low-salt wash buffer (20 mM Tris-HCl [pH 8.1], 150 mM NaCl, 2 mM EDTA, 0.1% SDS, 1% Triton X-100); high-salt wash buffer (20 mM Tris-HCl [pH 8.1], 500 mM NaCl, 2 mM EDTA, 0.1% SDS, 1% Triton X-100); LiCl wash buffer (10 mM Tris-HCl [pH 8.1], 0.25 M LiCl, 1% Nonidet P-40, 1% deoxycholate, 1 mM EDTA); and TE buffer (10 mM Tris-HCl [pH 8.1], 1 mM EDTA). Elution buffer (1% SDS, 0.1 M NaHCO_3_) was added and eluates were heated at 65°C overnight to reverse cross-linking. Eluates were treated with proteinase K for 1 hour at 45°C and DNA was purified by extraction in phenol/chloroform/isoamyl alcohol (25:24:1, v/v/v) and isopropanol precipitation. The pellet was washed with 70% ethanol and resuspended in water for qPCR analysis (see Supplemental Table 6 for antibody and other reagent sources).

### Animal studies.

Female nude mice (NCI Athymic NCr-nu/nu) and male C57L mice were purchased from Charles River and The Jackson Laboratory, respectively. Hep3B (8 × 10^6^) and Hepa1-6 (1 × 10^7^) cells were resuspended in PBS and Matrigel (1:1, vol/vol) and implanted subcutaneously in 6- to 8-week-old mice, and tumor volume was calculated as *V* = *abc* × 0.52. For Hep3B tumor studies, once tumors reached 100 to 150 mm^3^, mice were randomized into groups to receive daily intraperitoneal injection of either 32-134D or vehicle only (20% PEG-400, 10% Cremophor EL, 10% ethanol in PBS). For Hepa1-6 tumors, mice were randomized to receive treatment once tumors became palpable (tumor eradication experiment) or reached a volume of 200 mm^3^ (harvesting of tumors for flow cytometry and analysis of RNA and protein expression). Tumors were harvested 4 hours after the last treatment (see Supplemental Table 6 for reagent sources).

### Hematologic indices.

To analyze blood parameters, male C57BL/6J mice were treated with vehicle or 32-134D by daily intraperitoneal injection for 14 days. Blood was collected by cardiac puncture into EDTA-coated tubes for analysis of hematologic indices at the Johns Hopkins Mouse Phenotyping Core. Blood collected into noncoated tubes was allowed to clot for 2 hours at room temperature, centrifuged at 2000*g* for 20 minutes, and serum samples were subjected to EPO ELISA according to the manufacturer’s instructions (Supplemental Table 6). Organs including heart, liver, kidney, small intestine, colon, lungs, and brain were harvested, fixed in formalin for 24 hours, and embedded in paraffin for histopathological investigation. Hematoxylin and eosin staining was performed by the Johns Hopkins Reference Histology Services.

### Mouse pharmacokinetics.

Male C57BL/6J mice were administered 32-134D at 40 mg/kg as a single intraperitoneal injection. Mice (*n =* 3 per time point) were euthanized at 0.5, 1, 2, 4, 8, 16, and 24 hours after injection. Compound 32-134D was quantified in plasma by LC-MS/MS (see Supplemental Methods). Pharmacokinetic parameters were calculated from mean concentration-time data using noncompartmental methods in Phoenix WinNonlin version 8.3 (Certara). The C_max_ and time to C_max_ (*t*_max_) were the observed values. The AUC_last_ was calculated using the log-linear trapezoidal method. AUC was extrapolated to infinity (AUC_INF_) by dividing the last quantifiable concentration by the terminal disposition rate constant (λ_z_). The λ_z_ was determined from at least 3 points on the slope of the terminal phase of the concentration-time profile. The *t*_1/2_ was determined by dividing 0.693 by λ_z_. Cl/F was calculated by dividing the dose administered by AUC_INF_. V/F was calculated by dividing Cl/F by λ_z_. If the percentage AUC extrapolated was greater than 20% or the *r*^2^ of λ_z_ was less than 0.85, the AUC_INF_, Cl/F, *t*_1/2_, and V/F were not reported.

### Tumor immunohistochemistry.

Tumor samples were fixed in 10% formalin in phosphate buffer for 24 hours and placed in PBS the following day for paraffin embedding and sectioning. Anti-CD31 immunohistochemical staining, hematoxylin and eosin counterstaining, and whole-slide scanning were performed by NDB Bio (www.ndbbio.com). 

### Tumor ELISA.

Tumors were homogenized in ice-cold PBS supplemented with 1% Triton X-100 and PIs, centrifuged at 10,000*g* for 20 minutes to pellet debris, and the supernatant was collected for ELISA using commercial kits (Supplemental Table 6). Optical density was obtained at 450 nm (corrected for readings at 570 nm) using the VICTOR Nivo plate reader. Sample protein concentration was calculated by linear regression from a standard curve.

### Tumor cytokine mRNA assay.

Total RNA from Hepa1-6 tumors was analyzed using the RT^2^ Profiler PCR Array of Mouse Cytokines and Chemokines (Supplemental Table 6) according to the manufacturer’s instructions.

### Tumor flow cytometry.

Hepa1-6 tumors were digested with collagenase (1 mg/mL) at 37°C for 30 minutes and the resulting single-cell suspension was passed through a 70-μm cell strainer and washed twice with cold PBS. Cells were resuspended in FC buffer for subsequent flow cytometry analysis. Cells were stained with at least 2 antibodies (see Supplemental Table 6) to capture different immune cell populations. G-MDSCs: Alexa Fluor 405–conjugated anti-CD11b and FITC-conjugated anti-Ly6G; M-MDSCs: Alexa Fluor 405–conjugated anti-CD11b and FITC-conjugated anti-Ly6C; TAMs: Alexa Fluor 405–conjugated anti-CD11b and allophycocyanin-conjugated (APC-conjugated) anti-F4/80; dendritic cells: Alexa Fluor 405–conjugated anti-CD11b, FITC-conjugated anti-CD11c, and APC-conjugated anti-F4/80; cytotoxic NK cells: FITC-conjugated anti-CD3, APC-conjugated anti-NK1.1, and Alexa Fluor 405–conjugated anti-CD314; effector T cells: phycoerythrin-conjugated (PE-conjugated) anti-CD8A and Alexa Fluor 405–conjugated anti–IFN-γ; activated T cells: PE-conjugated anti-CD8A, FITC-conjugated anti-CD69, and APC-conjugated anti-CD44; regulatory T cells: APC-conjugated anti-CD4, FITC-conjugated anti-CD25, and PE-conjugated anti-FOXP3. Live cells were gated using side-scatter and forward-scatter plots, and data were acquired using FACSDiva software (Becton Dickinson). Cell populations were gated using unstained control and single stained cell samples. Data analysis was performed using FlowJo software.

### Statistics.

Data are expressed as mean ± SEM. For data presented in box-and-whisker plots, the top and bottom of the box represent the first and third quartiles, the line inside the box represents the median, and the top and bottom whiskers represent the maximum and minimum datum, respectively. Differences were considered statistically significant for *P* values of less than 0.05. Data were analyzed using the 2-tailed Mann-Whitney nonparametric *t* test for comparisons between 2 groups or ANOVA with the Tukey-Kramer test for multiple comparisons. The χ^2^ test was used to compare proportions. Analyses of association were performed using Spearman’s rank correlation test. Kaplan-Meier survival analysis (96) was performed using the log-rank test through an online tool (https://kmplot.com/analysis/), using the median mRNA expression level for stratification and OS at 3 years as the outcome measure for hazard ratio calculation.

### Study approval.

Protocols for animal studies were approved by the Johns Hopkins University Animal Care and Use Committee and were in accordance with the NIH *Guide for the Care and Use of Laboratory Animals* (97).

## Author contributions

SS and GLS conceived and designed research studies and experiments. SS, DJM, YH, EEW, SNL, ED, NMA, MAR, YL, and YY performed experiments and data analyses. SS, EEW, SNL, AMT, WJ and DD performed animal and flow cytometry experiments. GLS and SS interpreted data and wrote the manuscript. All authors revised and approved the final version of the manuscript.

## Supplementary Material

Supplemental data

## Version 1 05/02/2022

Electronic publication
