# Supplementary material for: HIF inhibitor 32-134D eradicates murine hepatocellular carcinoma in combination with anti-PD1 therapy
Source: J Clin Invest. 2022 May 2;132(9):e156774. doi: 10.1172/JCI156774 (PMC9057582; doi:10.1172/JCI156774)
Supplement: Supplemental data [file jci-132-156774-s078.pdf]

HIF inhibitor 32-134D eradicates murine hepatocellular carcinoma in combination with anti-PD1 therapy

Shaima Salman, David J. Meyers, Elizabeth E. Wicks, Sophia N. Lee, Emmanuel Datan, Aline M. Thomas, Nicole M. Anders, Yousang Hwang, Yajing Lyu, Yongkang Yang, Walter Jackson III, Dominic Dordai, Michelle A. Rudek, and Gregg L. Semenza

SUPPLEMENTAL MATERIAL

Supplemental Figures 1 – 9

Supplemental Tables 1 – 6

Supplemental Methods

Supplemental Figures

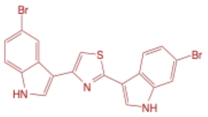

11-88
NSC-705870
IC50 2.9 μM

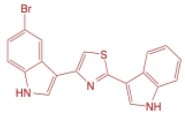

11-147B
IC50 2.9 μM

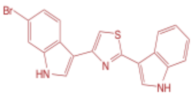

12-58
IC50 3.1 μM

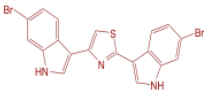

32-134C
IC50 2.3 μM

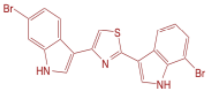

32-134D
IC50 2.5 μM

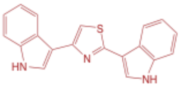

33-057A
IC50 2.9 μM

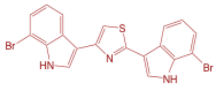

13-01
IC50 2.1 μM

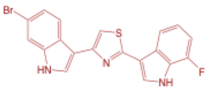

12-143
IC50 2.6 μM

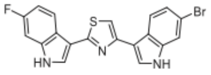

12-147A
IC50 2.5 μM

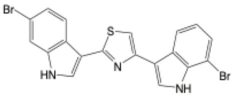

13-03
IC50 2.7 μM

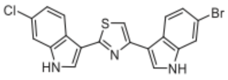

12-147B
IC50 3.1 μM

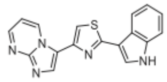

12-93F
IC50 2.5 μM

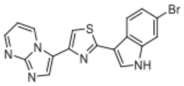

12-70C
IC50 2.3 μM

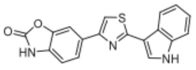

12-93A
IC50 2.4 μM

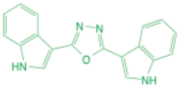

33-087
IC50 2.5 μM

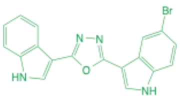

33-093B
IC50 2.7 μM

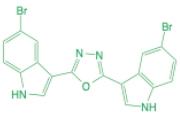

33-103B
IC50 2.9 μM

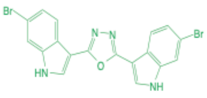

33-103C
IC50 2.8 μM

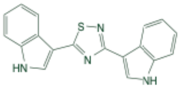

33-063
IC50 2.2 μM

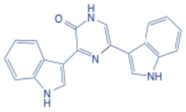

33-123
IC50 3.2 μM

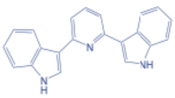

34-090
IC50 1.8 μM

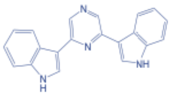

34-110
IC50 1.3 μM

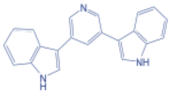

34-122
IC50 2.0 μM

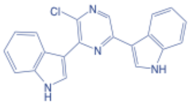

34-146
IC50 2.0 μM

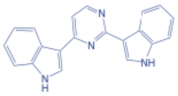

35-020
IC50 2.4 μM

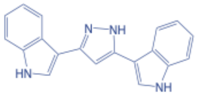

35-092
IC50 2.1 μM

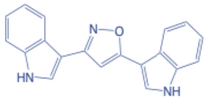

35-140
IC50 1.9 μM

Supplemental Figure 1. Chemical structure of compounds that inhibited HIF-dependent reporter gene expression in Hep3B-c1 cells. The concentration of each compound that inhibited reporter gene expression by 50% (IC_{50}) is shown.

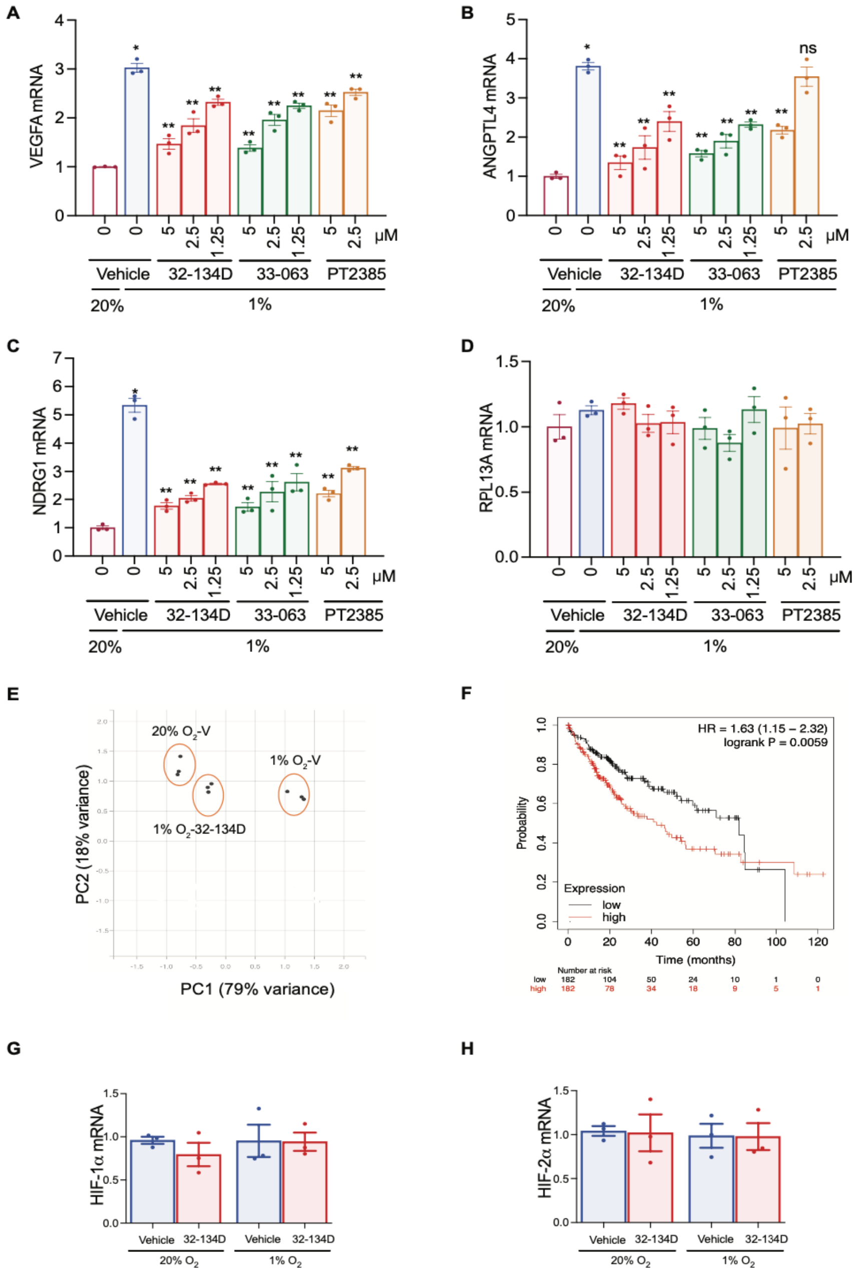

Supplemental Figure 2. Effect of 32-134D on gene expression in Hep3B cells. (A-D) Hep3B cells were exposed to 20% O_2 in the presence of vehicle (first bar; no fill) or 1% O_2 in the presence of: vehicle (blue bar) or the indicated concentration of 32-134D (bars with red fill) or 33-063 (bars with green fill), or PT2385 (bars with brown fill) for 24 hours and the indicated mRNAs were quantified by reverse transcription and quantitative real-time PCR (qPCR). Data are presented as mean $\pm$ SEM (n = 3). *$p < 0.05$ vs white; **$p < 0.01$ vs blue (ANOVA with Bonferroni post-test). (**E**) Principal component (PC) analysis of RNA-seq data. (**F**) Kaplan-Meier analysis was performed with HCC patients stratified based on intratumoral expression of a 15-gene HIF signature. The probability of overall survival is plotted on the y-axis. (**G-H**) Analysis of HIF-1α (**G**) and HIF-2α (**H**) mRNA expression in Hep3B cells exposed to vehicle or 32-134D at 20% or 1% O_2 for 24 hours.

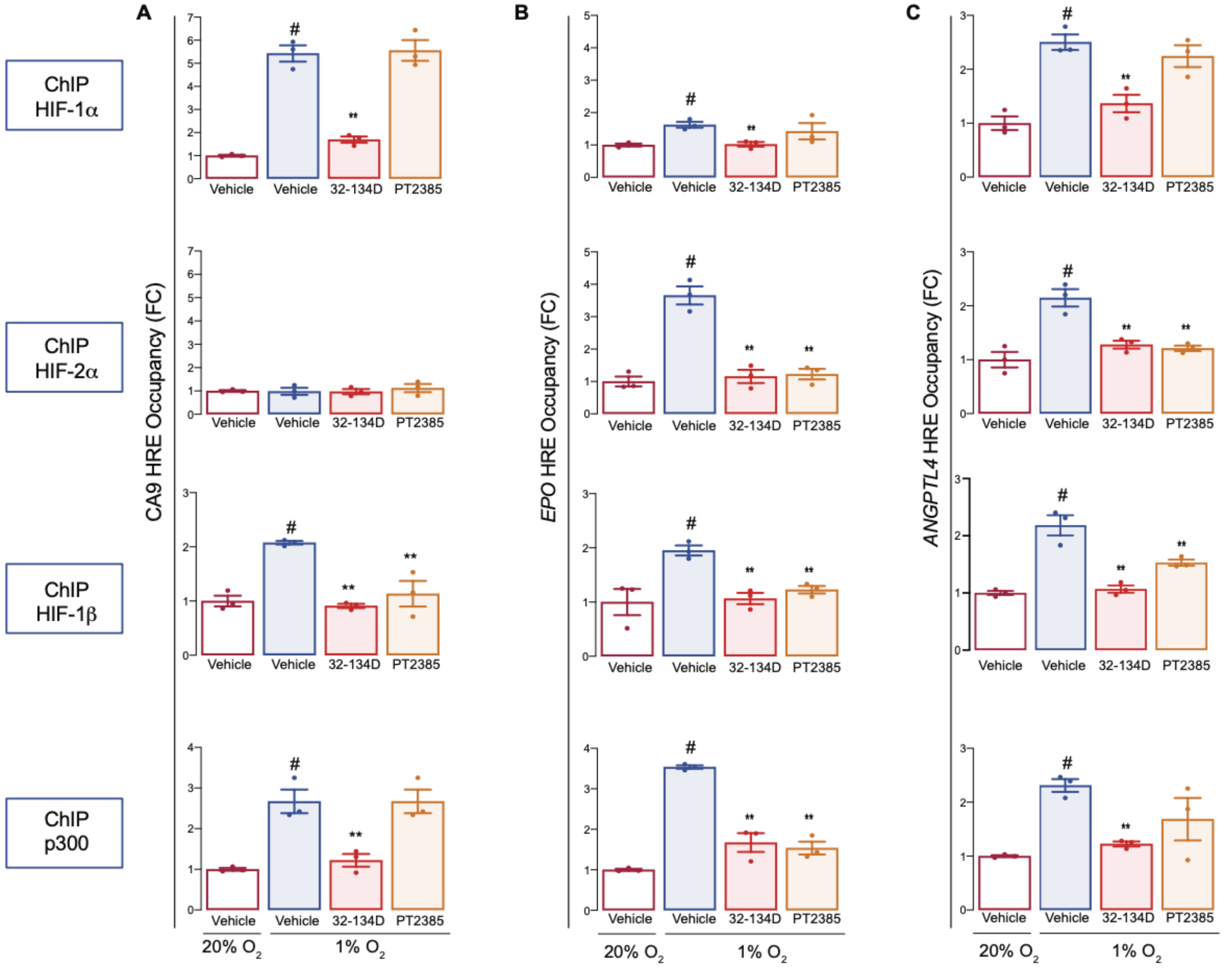

Supplemental Figure 3. Effect of 32-134D on hypoxia-induced occupancy of HREs by HIFs and P300. Hep3B cells were cultured for 16 hours at 20% O_2 in the presence of vehicle (V; white bars) or at 1% O_2 in the presence of vehicle (blue bars), 5 μM 32-134D (red bars) or 10 μM PT2385 (brown bars). Chromatin immunoprecipitation (ChIP) was performed using the indicated antibody (box), followed by qPCR using primers that flanked an HRE in the *CA9* (left column), *EPO* (middle column)*, or ANGPTL4* (right column) gene. Data are presented as mean $\pm$ SEM (n = 3) fold change (FC) normalized to white 20% O_2. $^{\#}p < 0.05$ vs white; $^{**}p < 0.01$ versus blue (ANOVA with Bonferroni post-test).

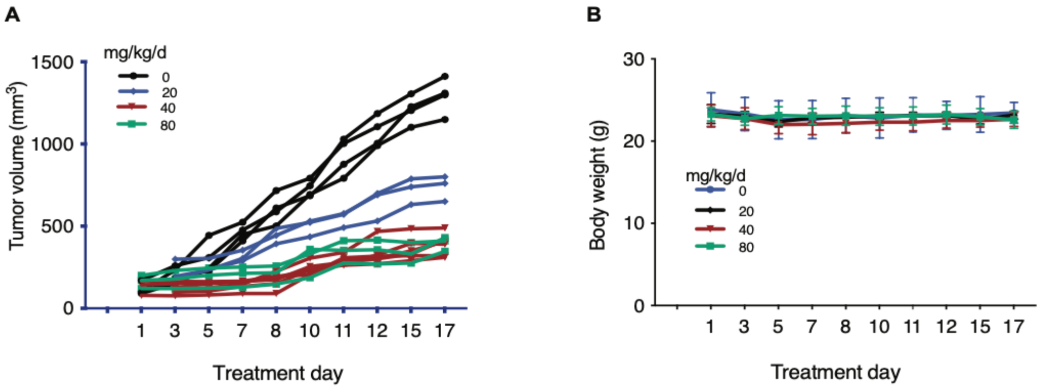

Supplemental Figure 4. Effect of 32-134D on tumor growth and body weight. Female nude mice received a subcutaneous injection of 8 x 10^6 Hep3B cells. When tumors reached a volume of 100-150 mm^3 (designated treatment day 1), the mice were randomized to receive a daily intraperitoneal injection of 32-134D at a dose of 0 (black), 20 (blue), 40 (red), or 80 (green) mg/kg. (**A**) Tumor volumes were determined every other day. Individual tumor growth curves are shown. (**B**) Body weights of tumor-bearing mice were measured every other data and are presented as mean $\underline{+}$ SEM (n = 3-4 per group).

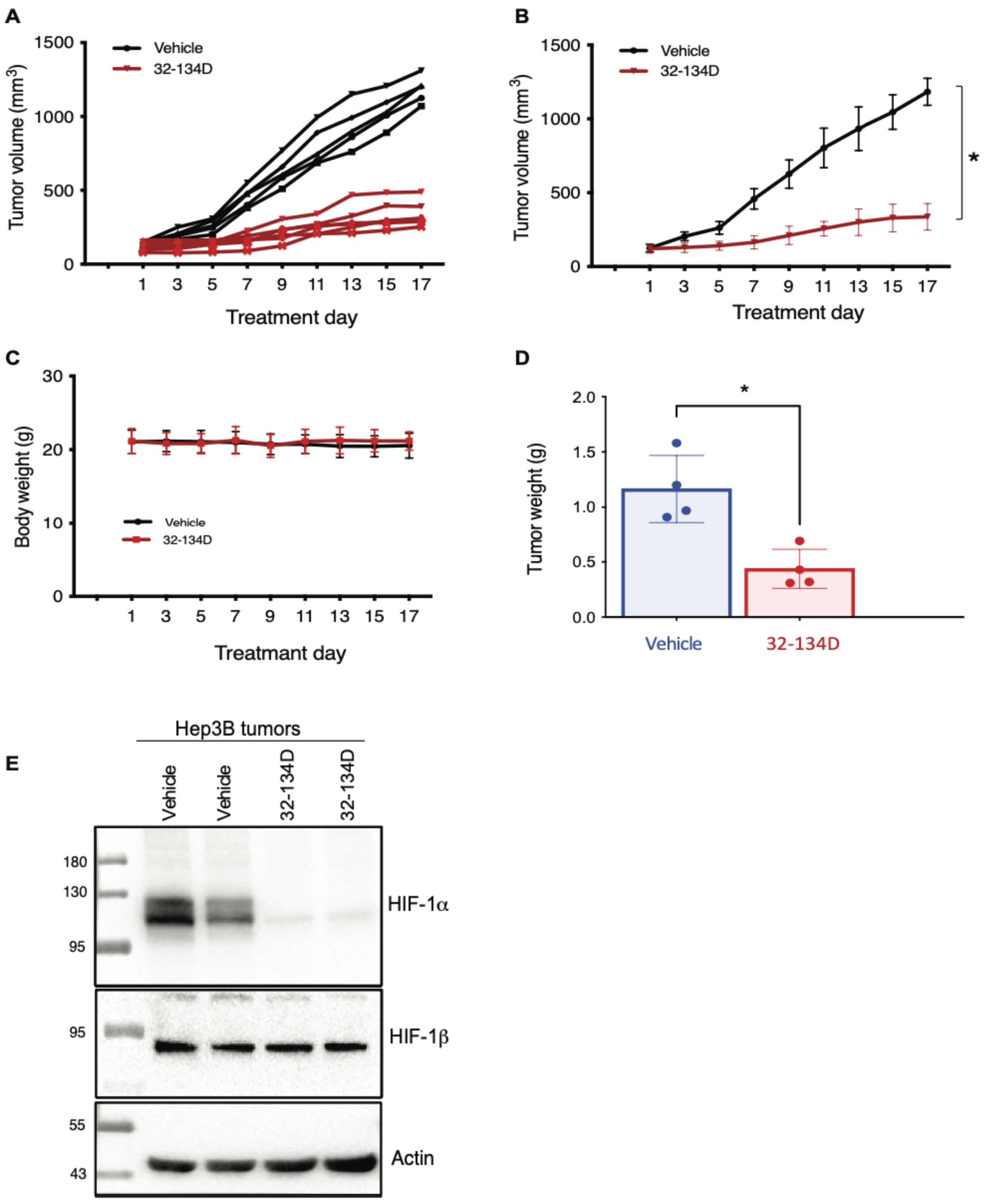

Supplemental Figure 5. Effect of 32-134D on tumor growth and body weight in additional mice. (**A-D**) Tumor bearing mice were treated with daily intraperitoneal injection of vehicle (black) or 32-134D (red; 40 mg/kg) and individual (**A**) and mean (**B**) tumor volumes as well as body weight (**C**) were determined every other day and mean tumor weights (**D**) were determined on day 17 (mean ± SEM, n =4). *P < 0.05 by ANOVA. (**E**) Nuclear extracts were prepared from tumors of vehicle-treated or 32-134D-treated mice and subjected to immunoblot assays using the indicated antibody.

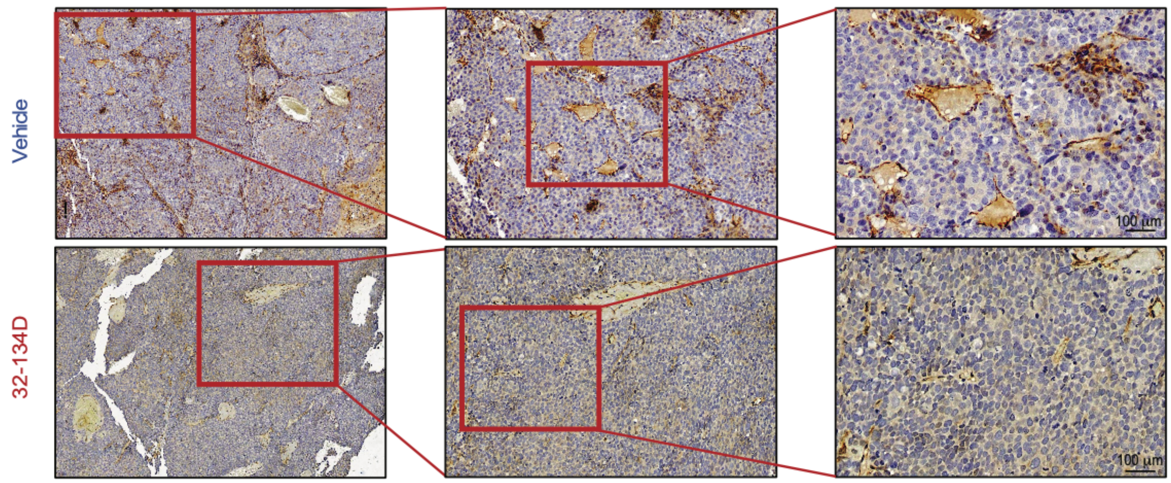

Supplemental Figure 6. Effect of 32-134D on tumor vascularization. Formalin-fixed and paraffin-embedded Hep3B tumor xenograft sections were subject to immunohistochemistry using an antibody against CD31 to identify vascular endothelial cells. Scale bar at bottom right indicates 100 µm.

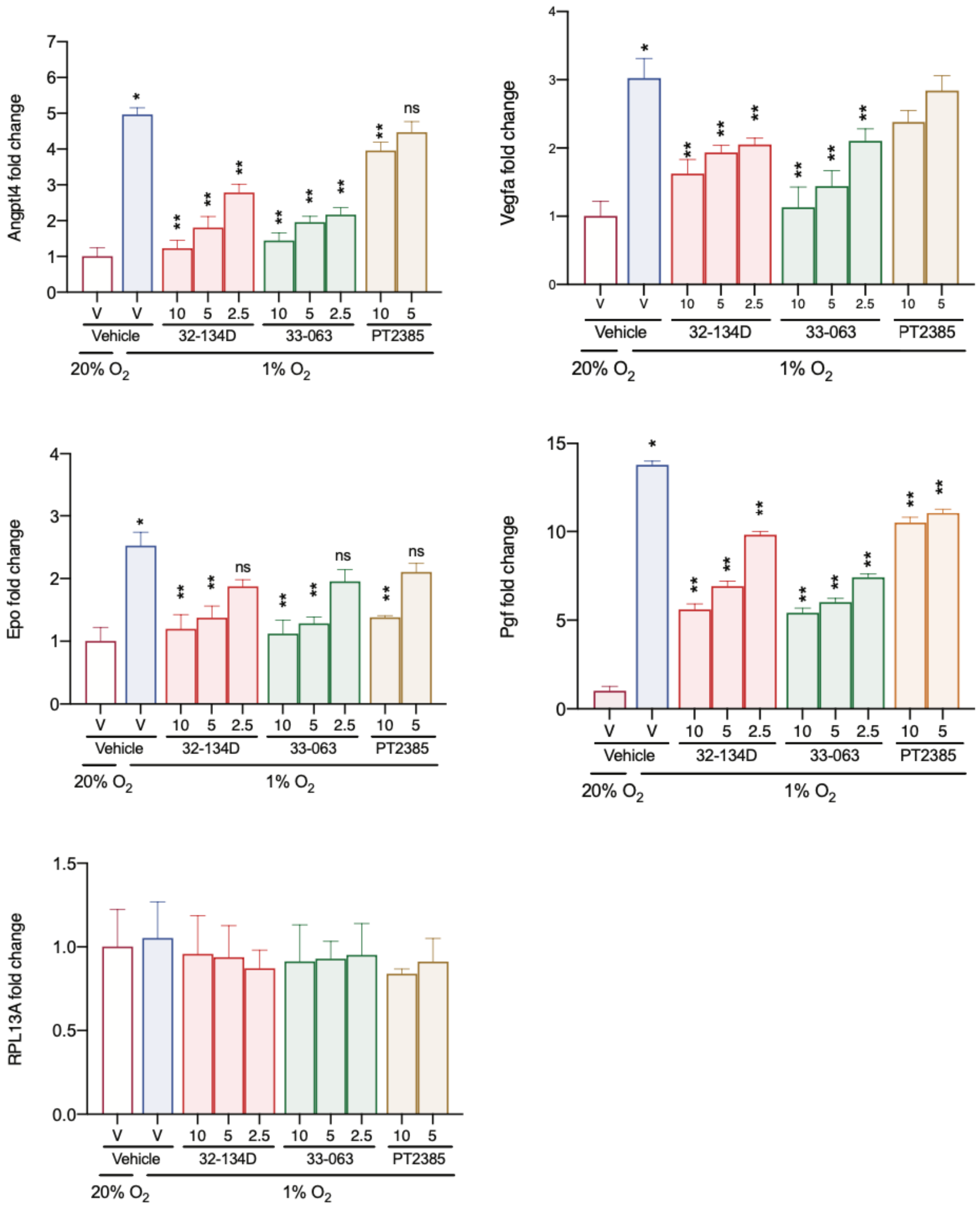

Supplemental Figure 7. Effect of 32-134D on gene expression in Hepa1-6 cells. Cells were exposed to 20% O_2 and vehicle (white bar) or 1% O_2 and vehicle (blue bar), 2.5-10 µM 32-134D (red bars), 2.5-10 µM 33-063 (green bars), or 5-10 µM PT2385 (brown bars) for 24 hours and mRNAs were quantified by RT-qPCR and normalized to white (mean ± SEM, n = 4). $^{*}p < 0.05$ vs white; $^{\#}p < 0.05$ vs blue (ANOVA with Bonferroni post-test).

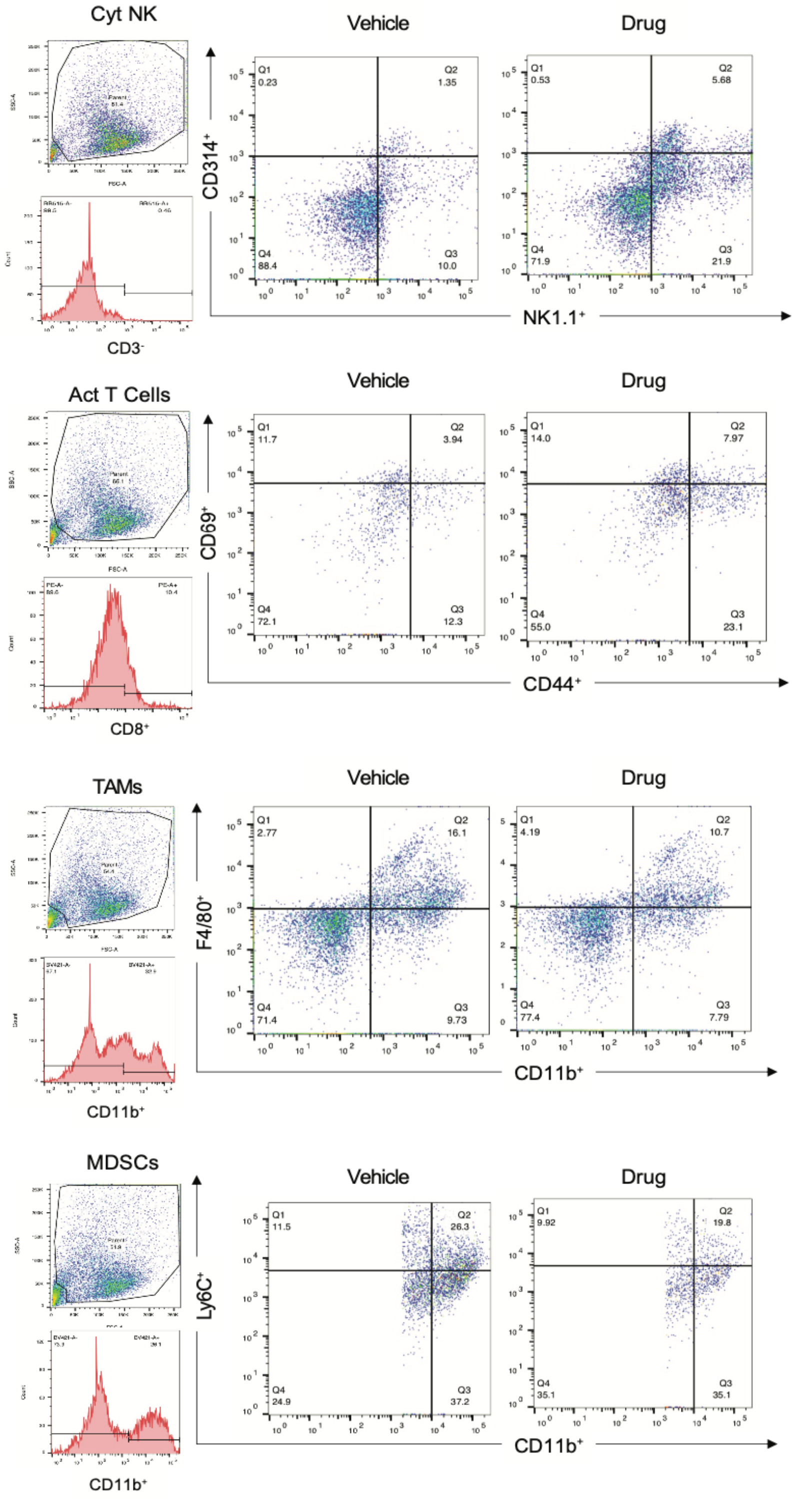

Supplemental Figure 8. Effect of 32-134D on Hepa1-6 tumor immune cell microenvironment. C57L mice were injected with Hepa1-6 HCC cells subcutaneously and when tumors reached a volume of 200 mm^3, the mice were treated with vehicle or 32-134D (40 mg/kg) by daily intraperitoneal injection for 8 days. Single cell suspensions prepared from each tumor were subjected to flow cytometry using fluorescent antibodies against the indicated cell surface proteins. Gating and representative data are shown.

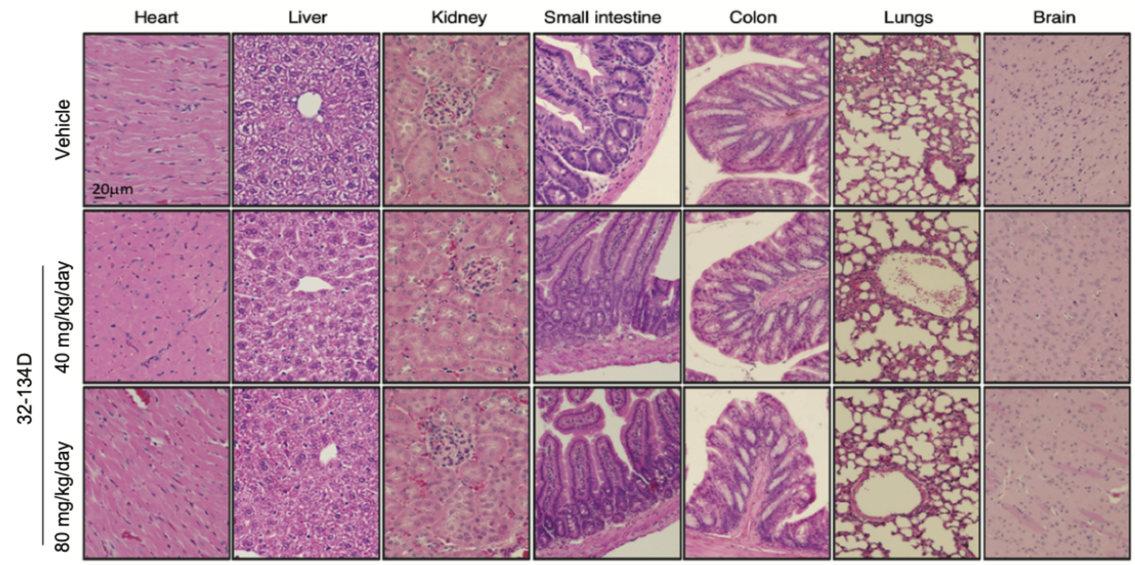

Supplemental Figure 9. Normal organ histology in mice treated with 32-134D. Mice were administered vehicle or 32-134D (40 or 80 mg/kg) by daily intraperitoneal injection for 14 days and tissue sections from the indicated organs were stained with hematoxylin and eosin. Scale bar = 20 μm.

Supplemental Tables

Supplemental Table 1. HIF signature in HCC. RNA-seq identified 15 known HIF target genes that were highly induced by exposure of Hep3B cells to 1% O_2 and inhibited by 32-134D. FC, fold change: ratio of mRNA expression in vehicle-treated cells at 1% vs 20% O_2.

mRNA	Log_2 FC*
ADM	5.14
ALDOA	2.98
ANGPTL4	5.39
BNIP3	3.85
CA9	4.34
EFNA1	4.08
ENO2	4.00
LDHA	3.41
P4HA1	4.21
PDK1	3.96
PFKFB4	5.11
PGK1	2.69
SLC2A1	4.18
STC2	3.95
TMCC1	2.83

Supplemental Table 2. Liver cancer mRNA expression associated with increased (HR < 1) or decreased (HR > 1) overall survival of HCC patients (n = 269) at 3 years by Kaplan-Meier analysis.

mRNA	HR*	p[**]
C5/HC[#]	0.56	3.9e-3
CCL2/CCL12[^]	0.67	4.5e-2
CD8A	0.52	1.3e-3
CD8B	0.62	1.7e-2
CXCL2	0.51	8.5e-4
CXCL9	0.63	2.0e-2
CXCL10	0.61	1.4e-2
KLRB1/NK1.1[&]	0.41	8.6e-6
KLRK1/CD314[##]	0.38	2.5e-6
CA9	2.49	6.0e-6
CXCL1	1.49	4.5e-2
EPO	1.67	9.8e-3
KITLG/SCF	2.28	4.8e-5
LDHA	2.35	2.6e-5
PGF	1.50	4.1e-2
SLC2A1/GLUT1	1.73	5.7e-3

*HR, hazard ratio; [**]p value, log rank test. [#]C5 is the human homolog of murine HC. [^]CCL2 is the human homolog of murine CCL12. [&]KLRB1 is the human homolog of murine NK1.1. [##]KLRK1 is also known as CD314.

Supplemental Table 3. Expression of cytokine mRNAs in tumors from mice treated with 32-134D (test group) or vehicle (control group) as determined by an RT-qPCR array.

			Fold Change	T-TEST	Fold Up- or Down-Regulation	Y axis	X axis
	Symbol	Well	Test Group /Control Group	p value	Test Group /Control Group	(-Log10(pValue))	(Log2(fold change)
1	Adipoq	A01	0.43	0.0159	-2.31	1.8	-1.21
2	Bmp2	A02	0.51	0.1529	-1.95	0.8	-0.96
3	Bmp4	A03	0.64	0.3124	-1.56	0.5	-0.64
4	Bmp6	A04	0.44	0.0138	-2.27	1.9	-1.18
5	Bmp7	A05	0.70	0.4516	-1.43	0.3	-0.51
6	Ccl1	A06	0.63	0.4314	-1.58	0.4	-0.66
7	Ccl11	A07	1.03	0.7782	1.03	0.1	0.04
8	Ccl12	A08	4.11	0.0251	4.11	1.6	2.04
9	Ccl17	A09	0.68	0.4482	-1.47	0.3	-0.56
10	Ccl19	A10	0.54	0.0011	-1.86	3.0	-0.89
11	Ccl2	A11	1.35	0.2081	1.35	0.7	0.44
12	Ccl20	A12	0.42	0.0056	-2.41	2.3	-1.27
13	Ccl22	B01	0.31	0.0027	-3.18	2.6	-1.67
14	Ccl24	B02	1.21	0.4075	1.21	0.4	0.27
15	Ccl3	B03	1.06	0.7500	1.06	0.1	0.09
16	Ccl4	B04	0.72	0.3408	-1.39	0.5	-0.47
17	Ccl5	B05	0.92	0.5575	-1.09	0.3	-0.13
18	Ccl7	B06	1.03	0.7626	1.03	0.1	0.04
19	Cd40lg	B07	0.52	0.0452	-1.91	1.3	-0.93
20	Cd70	B08	0.24	0.0010	-4.19	3.0	-2.07
21	Cntf	B09	0.38	0.0275	-2.61	1.6	-1.39
22	Csf1	B10	1.02	0.8491	1.02	0.1	0.03
23	Csf2	B11	0.38	0.0024	-2.64	2.6	-1.40
24	Csf3	B12	0.50	0.0033	-1.99	2.5	-0.99
25	Ctf1	C01	0.19	0.0005	-5.18	3.3	-2.37
26	Cx3cl1	C02	0.53	0.0005	-1.89	3.3	-0.92
27	Cxcl1	C03	0.67	0.0072	-1.49	2.1	-0.57
28	Cxcl10	C04	1.44	0.0484	1.44	1.3	0.53
29	Cxcl11	C05	0.45	0.0055	-2.24	2.3	-1.16
30	Cxcl12	C06	2.26	0.1827	2.26	0.7	1.17
31	Cxcl13	C07	0.39	0.0003	-2.57	3.5	-1.36
32	Cxcl16	C08	0.61	0.0540	-1.65	1.3	-0.72
33	Cxcl3	C09	0.87	0.1486	-1.14	0.8	-0.19
34	Cxcl5	C10	1.05	0.7156	1.05	0.1	0.07
35	Cxcl9	C11	1.64	0.0001	1.64	4.0	0.71
36	Fasl	C12	0.59	0.0019	-1.70	2.7	-0.77
37	Gpi1	D01	0.64	0.0398	-1.57	1.4	-0.65
38	Hc	D02	1.77	0.0180	1.77	1.7	0.82
39	Ifna2	D03	0.26	0.0011	-3.85	3.0	-1.95
40	Ifng	D04	1.63	0.0015	1.63	2.8	0.70
41	Il10	D05	0.23	0.0012	-4.42	2.9	-2.14
42	Il11	D06	0.24	0.0043	-4.12	2.4	-2.04
43	Il12a	D07	0.48	0.0138	-2.07	1.9	-1.05
44	Il12b	D08	0.29	0.0029	-3.51	2.5	-1.81
45	Il13	D09	0.32	0.0071	-3.14	2.1	-1.65
46	Il15	D10	0.84	0.3524	-1.19	0.5	-0.25
47	Il16	D11	0.53	0.0145	-1.90	1.8	-0.93
48	Il17a	D12	0.33	0.0045	-3.00	2.3	-1.59
49	Il17f	E01	0.75	0.0926	-1.33	1.0	-0.41
50	Il18	E02	0.89	0.4793	-1.13	0.3	-0.17
51	Il1a	E03	0.68	0.4472	-1.47	0.3	-0.56
52	Il1b	E04	0.34	0.2747	-2.92	0.6	-1.55
53	Il1rn	E05	0.68	0.0957	-1.47	1.0	-0.56
54	Il2	E06	0.30	0.0190	-3.37	1.7	-1.75
55	Il21	E07	0.29	0.0098	-3.46	2.0	-1.79
56	Il22	E08	0.37	0.0042	-2.67	2.4	-1.42
57	Il23a	E09	0.44	0.1786	-2.25	0.7	-1.17
58	Il24	E10	0.43	0.0232	-2.34	1.6	-1.23
59	Il27	E11	0.46	0.1550	-2.19	0.8	-1.13
60	Il3	E12	0.36	0.0041	-2.77	2.4	-1.47
61	Il4	F01	0.34	0.0010	-2.91	3.0	-1.54
62	Il5	F02	0.42	0.0710	-2.39	1.1	-1.26
63	Il6	F03	0.34	0.0209	-2.95	1.7	-1.56
64	Il7	F04	0.97	0.9540	-1.04	0.0	-0.05
65	Il9	F05	0.27	0.0015	-3.69	2.8	-1.88
66	Lif	F06	0.40	0.0807	-2.48	1.1	-1.31
67	Lta	F07	0.34	0.0331	-2.96	1.5	-1.57
68	Ltb	F08	0.63	0.0784	-1.58	1.1	-0.66
69	Mif	F09	1.12	0.5514	1.12	0.3	0.17
70	Mstn	F10	0.21	0.0941	-4.85	1.0	-2.28
71	Nodal	F11	0.29	0.0009	-3.43	3.1	-1.78
72	Osm	F12	0.38	0.0329	-2.65	1.5	-1.40
73	Pf4	G01	0.68	0.4216	-1.47	0.4	-0.56
74	Ppbp	G02	0.64	0.1634	-1.56	0.8	-0.65
75	Spp1	G03	1.71	0.0649	1.71	1.2	0.77
76	Tgfb2	G04	0.74	0.2996	-1.36	0.5	-0.44
77	Thpo	G05	0.55	0.3920	-1.82	0.4	-0.87
78	Tnf	G06	0.25	0.0181	-4.00	1.7	-2.00
79	Tnfrsf11b	G07	0.25	0.0007	-4.07	3.2	-2.02
80	Tnfsf10	G08	1.02	0.7133	1.02	0.1	0.03
81	Tnfsf11	G09	0.38	0.1108	-2.61	1.0	-1.39
82	Tnfsf13b	G10	0.88	0.6833	-1.13	0.2	-0.18
83	Vegfa	G11	0.40	0.0058	-2.52	2.2	-1.33
84	Xcl1	G12	1.47	0.2829	1.47	0.5	0.56

Supplemental Table 4. HIF-regulated genes that may contribute to immunosuppression in HCC.

Gene	Mechanism	Reference(s)
CA9	Creates an acidic extracellular milieu that inhibits T cells	74, 80-82
CD47	Blocks macrophage engulfment of cancer cells	S1
CD70	Induces immune cell apoptosis	70-72
CD73	Converts AMP to adenosine	S2
CXCL1	Induces recruitment of TAMs	S3
ENTPD1	Encodes CD39, converts ATP to AMP	S2
HAVCR2	Encodes TIM3, induces T cell exhaustion	S4, S5
lL4	Induces M2 polarization of TAMs	S6-S8
IL6	Activates MDSCs; induces M2 polarization of TAMs	S9-S11
IL10	Maintains Tregs; inhibits APCs; induces TAM polarization	S12, S13
IL13	Induces M2 polarization of TAMs	S6, S7
IL22	Enhances production of IL10 and TGF-β by cancer cells	S8
LDHA	Produces lactic acid to inhibit immune cell function	73-75, 77-79
PDL1	Induces T cell exhaustion	11
PGF	Promotes polarization of TAMs; inhibits DC maturation	S8
SLC2A1	Cancer cells compete with immune cells for glucose	76
VEGFA	Induces recruitment of MDSCs, TAMs, and Tregs	S8
	Inhibits dendritic cell maturation/activation	S14
VTCN1	Encodes B7H4, induces T cell exhaustion	S15

Supplemental References

S1. Chen J, et al. Macrophages induce CD47 upregulation via IL-6 and correlate with poor survival in hepatocellular carcinoma. *Oncoimmunology*. 2019;8(11):1652540.

S2. Pang L, et al. Plasmacytoid dendritic cells recruited by HIF-1α/eADO/ADORA1 signaling induce immunosuppression in hepatocellular carcinoma. *Cancer Lett*. 2021;522:80-92.

S3. Miyake M, et al. CXCL1-mediated interaction of cancer cells with tumor-associated macrophages and cancer-associated fibroblasts promotes tumor progression in human bladder cancer. *Neoplasia*. 2016;18(10):636-646.

S4. Hakemi MG, et al. The role of TIM-3 in hepatocellular carcinoma: a promising target for immunotherapy? *Front Oncol*. 2020;10:601661

S5. Leone P, et al. The evolving role of immune checkpoint inhibitors in hepatocellular carcinoma treatment. *Vaccines*. 2021;9(5):532.

S6. Deng L, et al. The role of tumor-associated macrophages in primary hepatocellular carcinoma and its related targeting therapy. *Int J Med Sci*. 2021;18(10): 2109-2116.

S7. Han C, et al. The mechanism of lncRNA-CRNDE in regulating tumor-associated macrophage M2 polarization and promoting tumor angiogenesis. *J Cell Mol Med*. 2021;25(9):4235-4247.

S8. Voron T, et al. Control of the immune response by pro-angiogenic factors. *Front Oncol*. 2014;4:70.

S9. Lin Y, et al. Chemerin has a protective role in hepatocellular carcinoma by inhibiting the expression of IL-6 and GM-CSF and MDSC accumulation. *Oncogene*. 2017;36:3599-3608.

S10. Xu M, et al. Interactions between interleukin-6 and myeloid-derived suppressor cells drive the chemoresistant phenotype of hepatocellular carcinoma. *Exp Cell Res*. 2017;351:142-149.
S11. Yang J, Xing Z. Lingustilide counteracts carcinogenesis and hepatocellular carcinoma cell-evoked macrophage M2 polarization by regulating yes-associated protein mediated interleukin-6 secretion. *Exp Biol*. 2021;246(17):1928-1937.
S12. Bogdan C, et al. Macrophage deactivation by interleukin 10. *J Exp Med*. 1991;174:1549.
S13. Murai M, et al. Interleukin 10 acts on regulatory T cells to maintain expression of the transcription factor Foxp3 and suppressive function in mice with colitis. *Nat Immunol*. 2009;10:1178-1184.
S14. Yang J, et al. Targeting VEGF/VEGFR to modulate antitumor immunity. *Front Immunol*. 2018;9:978.
S15. MacGregor HL, Ohashi PS. Molecular pathways: evaluating the potential for B7-H4 as an immunoregulatory target. *Clin Cancer Res*. 23(12):2934-2941.

Supplemental Table 5. Forward (F) and reverse (R) PCR primers for amplification of human (H) and mouse (m) sequences.

Oligonucleotide	Sequence (5' to 3')	Source	Application
H-ANGPTL4-F	GGACACGGCCTATAGCCTG	Sigma	RT-qPCR
H-ANGPTL4-R	CTCTTGGCGCAGTTCTTGTC	Sigma	RT-qPCR
H-VEGFA-F	AGGGCAGAATCATCACGAAGT	Sigma	RT-qPCR
H-VEGFA-R	AGGGTCTCGATTGGATGGCA	Sigma	RT-qPCR
H-CA9-F	TCTCGTTTCCAATGCACGTACAGC	Sigma	RT-qPCR
H-CA9-R	AGTGACAGCAGCAGTTGCACAGT	Sigma	RT-qPCR
H-EPO-F	GGAGGCCGAGAATATCACGAC	Sigma	RT-qPCR
H-EPO-R	CCCTGCCAGACTT CTACGG	Sigma	RT-qPCR
H-NDRG1-F	CTCCTGCAAGAGTTTGATGTCC	Sigma	RT-qPCR
H-NDRG1-R	CATGCCGATGTCATGGTAGG	Sigma	RT-qPCR
H-SDF-1-F	ATTCTCAACACTCCAAACTGTGC	Sigma	RT-qPCR
H-SDF-1-R	ACTTTAGCTTCGGGTCAATGC	Sigma	RT-qPCR
H-PGF-F	GAACGGCTCGTCAGAGGTG	Sigma	RT-qPCR
H-PGF-R	ACAGTGCAGATTCTCATCGCC	Sigma	RT-qPCR
H-CD73-F	GCCTGGGAGCTTACGATTTTG	Sigma	RT-qPCR
H-CD73-R	TAGTGCCCTGGTACTGGTCG	Sigma	RT-qPCR
H-CD47-F	AGAAGGTGAAACGATCATCGAGC	Sigma	RT-qPCR
H-CD47-R	CTCATCCATACCACCGGATCT	Sigma	RT-qPCR
H-PDL1-F	CCAGGATGGTTCTTAGACTCCC	Sigma	RT-qPCR
H-PDL1-R	TTTAGCACGAAGCTCTCCGAT	Sigma	RT-qPCR
H-SCF-F	AATCCTCTCGTCAAAACTGAAGG	Sigma	RT-qPCR
H-SCF-R	CCATCTCGCTTATCCAACAATGA	Sigma	RT-qPCR
H-RPL13A-F	CTCAAGGTCGTGCGTCTG	Sigma	RT-qPCR
H-RPL13A-R	TGGCTTTCTCTTTCCTCTTCTC	Sigma	RT-qPCR
H-18S-rRNA-F	GTAACCCGTTGAACCCCATT	Sigma	RT-qPCR
H-18S-rRNA-R	CCATCTCGGCTTATCCAACAATGA	Sigma	RT-qPCR
m-Angptl4-F	CATCCTGGGACGAGATGAACT	Sigma	RT-qPCR
m-Angptl4-R	TGACAAGCGTTACCACAGGC	Sigma	RT-qPCR
m-Epo-F	ACTCTCCTTGCTACTGATTCCT	Sigma	RT-qPCR
m-Epo-R	ATCGTGACATTTTCTGCCTCC	Sigma	RT-qPCR
m-Pgf-F	TCTGCTGGGAACAACTCAACA	Sigma	RT-qPCR
m-Pgf-R	GTGAGACACCTCATCAGGGTAT	Sigma	RT-qPCR
m-Rpl13a-F	GGGCAGGTTCTGGTATTGGAT	Sigma	RT-qPCR
m-Rpl13a-R	GGCTCGGAAATGGTAGGGG	Sigma	RT-qPCR
m-18S-F	GTAACCCGTTGAACCCCATT	Sigma	RT-qPCR
m-18S-R	CCATCCAATCGGTAGTAGCG	Sigma	RT-qPCR
m-B7h4-F	CTTTGGCATTTCAGGCAAGCA	Sigma	RT-qPCR

m-B7h4-R	TGATGTCAGGTTCAAAAGTGCAG	Sigma	RT-qPCR
m-Ca9-F	TGCTCCAAGTGTCTGCTCAG	Sigma	RT-qPCR
m-Ca9-R	CAGGTGCATCCTCTTCACTGG	Sigma	RT-qPCR
m-Cd47-F	TGGTGGGAAACTACACTTGCG	Sigma	RT-qPCR
m-Cd47-R	CGTGCGGTTTTTCAGCTCTAT	Sigma	RT-qPCR
m-Cd70-F	TGTAGCGGACTACTCAGTAAGC	Sigma	RT-qPCR
m-Cd70-R	TGGGGTCCTTCCGAGGAAC	Sigma	RT-qPCR
m-Cd73-F	CCTGCACACAAACGACGTG	Sigma	RT-qPCR
m-Cd73-R	CTGGTCTCCGGCATCCAAAA	Sigma	RT-qPCR
m-Cxcl1-F	ACTGCACCCAAACCGAAGTC	Sigma	RT-qPCR
m-Cxcl1-R	TGGGGACACCTTTTAGCATCTT	Sigma	RT-qPCR
m-Entpd1-F	AAGGTGAAGAGATTTTGCTCCAA	Sigma	RT-qPCR
m-Entpd1-R	TTTGTTCTGGGTCAGTCCCAC	Sigma	RT-qPCR
m-Il6-F	TAGTCCTTCCTACCCCAATTTCC	Sigma	RT-qPCR
m-Il6-R	TTGGTCCTTAGCCACTCCTTC	Sigma	RT-qPCR
m-Il10-F	GCTCTTACTGACTGG	Sigma	RT-qPCR
m-Il11-F	TGTTCTCCTAACCCGATCCCT	Sigma	RT-qPCR
m-Il11-R	CAGGAAGCTGCAAAGATCCCA	Sigma	RT-qPCR
m-Il22-F	ATGAGTTTTTCCCTTATGGGGAC	Sigma	RT-qPCR
m-Il22-R	GCTGGAAGTTGGACACCTCAA	Sigma	RT-qPCR
m-Ldha-F	TGTCTCCAGCAAAGACTACTGT	Sigma	RT-qPCR
m-Ldha-R	GACTGTACTTGACAATGTTGGGA	Sigma	RT-qPCR
m-Pdl1-F	GACCAGCTTTTGAAGGGAATG	Sigma	RT-qPCR
m-Pdl1-R	CTGGTTGATTTTGCGGTATGG	Sigma	RT-qPCR
m-Slc2a1-F	CAGTTCGGCTATAACACTGGTG	Sigma	RT-qPCR
m-Slc2a1-R	GCCCCCGACAGAGAAGATG	Sigma	RT-qPCR
m-Tim3-F	TCAGGTCTTACCCTCAACTGTG	Sigma	RT-qPCR
m-Tim3-R	GGGCAGATAGGCATTTTTACCA	Sigma	RT-qPCR
m-Vegfa-F	CTGCCGTCCGATTGAGACC	Sigma	RT-qPCR
m-Vegfa-R	CCCCTCCTTGTACCACTGTC	Sigma	RT-qPCR
H-ANGPTL4-HRE-F	ATTTGCTGTCCTGGCATC	Sigma	ChIP-qPCR
H-ANGPTL4-HRE-R	CCAGCTCATTCTCTGGAATC	Sigma	ChIP-qPCR
H-CA9-HRE-F	GACAAACCTGTGAGACTTTGGCTCC	Sigma	ChIP-qPCR
H-CA9-HRE-R	AGTGACAGCAGCAGTTGCACAGTG	Sigma	ChIP-qPCR
H-EPO-HRE-F	GCCCTACGTGCTGTCTCACAC	Sigma	ChIP-qPCR
H-EPO-HRE-F	CCTTGATGACAATCTCAGCGC	Sigma	ChIP-qPCR
H-PDK1-HRE-F	CGCGTTTGGATTCCGTG	Sigma	ChIP-qPCR
H-PDK1-HRE-R	CCAGTTATAATCTGCCTTCCCTATTATC	Sigma	ChIP-qPCR
H-RPL13A-F	GAGGCGAGGGTGATAGAG	Sigma	ChIP-qPCR
H-RPL13A-R	ACACACAAGGGTCCAATTC	Sigma	ChIP-qPCR

Supplemental Table 6. Antibodies for ChIP, immunoblot (IB) assays and ELISAs for quantification of human (h) and mouse (m) proteins; antibodies for flow cytometry (FC) and in vivo studies (IV), experimental models, chemicals, peptides, and other reagents.

**Target protein**	**Catalog number**	**Vendor**	**Application**
h-HIF-1α	NB100-479	Novus Biologicals	IB, ChIP
h-HIF-1β	NB100-110	Novus Biologicals	ChIP
h-HIF-2α	NB100-122	Novus Biologicals	IB, ChIP
h-p300	NB500-161	Novus Biologicals	ChIP
m-β-Actin	Sc-47778	Santa Cruz Biotechnology	IB
m-Cxcl1	DY453	Novus Biologicals	ELISA
m-Cxcl2	MM200	Novus Biologicals	ELISA
m-Cxcl9	MCX900	Novus Biologicals	ELISA
m-Cxcl10	DY466	Novus Biologicals	ELISA
m-Il22	M2200	Novus Biologicals	ELISA
m-Il6	M6000B	Novus Biologicals	ELISA
m-Il10	DY417	Novus Biologicals	ELISA
m-Vegfa	MMV00	Novus Biologicals	ELISA
h-EPO	DEP00	Novus Biologicals	ELISA
h-SCF	DCK00	Novus Biologicals	ELISA
h-SDF-1α	DSA00	Novus Biologicals	ELISA
h-VEGFA	DVE00	Novus Biologicals	ELISA
m-EPO	MEP00B	Novus Biologicals	ELISA
m-Cd3	FAB4841G	Novus Biologicals	FC
m-Cd4	FAB554A	Novus Biologicals	FC
m-Cd8A	NBP1-49045-PE	Novus Biologicals	FC
m-Cd11b	NB110-89474-AF405	Novus Biologicals	FC
m-Cd11c	NB110-40766-AF488	Novus Biologicals	FC
m-Cd25	NBP2-27425-AF488	Novus Biologicals	FC
m-Cd44	NBP1-47386-APC	Novus Biologicals	FC
m-Cd69	NBP1-28011-AF488	Novus Biologicals	FC
m-Cd314	FAB 1547V	Novus Biologicals	FC
m-F4/80	NB600-404-APC	Novus Biologicals	FC
m-Foxp3	NB100-39002-PE	Novus Biologicals	FC
m-Ifng	IC485V	Novus Biologicals	FC
m-Ly6c	NBP1-28046-AF488	Novus Biologicals	FC
m-Ly6g	FAB1037A	Novus Biologicals	FC
m-Nk1.1	NB100-77528-APC	Novus Biologicals	FC
Rat IgG2a, κ, anti-m-PD1	BE0089	Bio X Cell	IV
Rat IgG2a isotype control	Bio X Cell	Bio X Cell	IV

Hep3B human hepatocellular carcinoma cell line	HB-8064	ATCC	Cell culture
Hepa1-6 mouse hepatoma cell line	CRL-1830	ATCC	Cell culture
Dulbecco's modified Eagle's medium	10-013-CV	ThermoFisher	Cell culture
FBS	100-106 BenchMark FBS	GeminiBio	Cell culture
Penicillin-Streptomycin	P0781	Sigma	Cell culture
Matrigel Matrix Basement Membrane	354234	Westnet	IV
TRIzol Reagent	15596026	Invitrogen	RNA
CREMOPHOR EL STERILIZED	238470-1SET	Sigma	IP Formulation
PEG-400	PX1286B-2	Sigma	IP Formulation
Ethanol	1009861000	Sigma	IP Formulation
ECL Prime reagent	SKU# GERPN2232	GE Healthcare	IB
Collagenase type I	SCR103	Sigma Aldrich	Tumor study
High-Capacity RNA-to-cDNA Kit	4387406	Applied Biosystem	RNA
RNase-Free DNase Set	79254	Qiagen	RNA
iQ SYBR Green Supermix	1708884	BioRad	qPCR
RT2 ProfilerTM PCR Array of Mouse Cytokines & Chemokines	330231 PAMM-150ZA	Qiagen	RT2-qPCR assay
Dual Luciferase Reporter Assay System	E1960	Promega	Luciferase reporter assay
HEPES	15-630-080	ThermoFisher	IB
Protease Inhibitor	P8340	Sigma	IB
Triton X-100	11332481001	Sigma	IB
EDTA	60-00-4	Sigma	IB
Tris-HCl	10812846001	Sigma	IB
glycerol	G5516	Sigma	IB
salmon sperm DNA/protein A agarose slurry	16-157	Sigma	ChIP
37% formaldehyde	F79-500	Sigma	ChIP
SDS	151-21-3	Sigma	IB, ChIP
Sodium deoxycholate	302-95-4	Sigma	ChIP
UltraPure™ Phenol:Chloroform:Isoamyl Alcohol (25:24:1, v/v)	15593031	ThermoFisher	ChIP
Nonidet P-40	J19628.K2	ThermoFisher	ChIP

Supplemental Methods

General chemistry procedures. All commercially available reagents and solvents were used without further purification unless otherwise stated. Automated flash chromatography was performed on a Teledyne Isco CombiFlash Rf+ or Grace Reveleris using Teledyne Isco or Grace/Buchi flash silica and/or C18 cartridges. Spectra were recorded on a Bruker Avance-III spectrometer (^{1}H NMR at 500 MHz and ^{13}C NMR at 125 MHz) at 296 K in $CDCl_3$ (^{1}H NMR referenced to internal standard tetramethylsilane 0 ppm, ^{13}C NMR referenced to 77.00 ppm), d6-DMSO (^{1}H NMR referenced to 2.50 ppm, ^{13}C NMR referenced to 39.510 ppm). Analytical LC-MS was performed using Agilent 1260 equipped with autosampler (Agilent Poroshell 120 column (50 x 3.0 mm I.D., 2.7 µm); 0.05% TFA in water/acetonitrile gradient; UV detection at 220 and 254 nm) and electrospray ionization. Unless otherwise noted, all final compounds showed purity greater than 95% at 215 and 254 nm using this method.

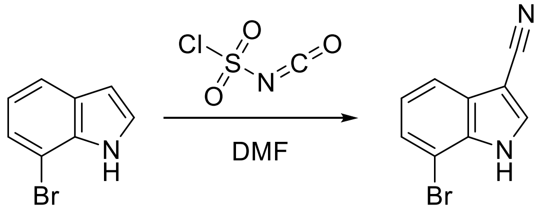

Synthesis of 7-bromo-1H-indole-3-carbonitrile. This procedure is a slight modification to that reported by Vorbruggen and Krolikiewicz (1). To a rapidly stirred solution of 7-bromoindole (5.0 g, 25.5 mmol, 1.0 equiv.) in anhydrous DMF (17 ml) was added chlorosulfonyl isocyanate (2.4 ml, 28.1 mmol, 1.1 equiv.) dropwise while cooling with an ice water bath. Once addition was complete, the ice water bath was allowed to equilibrate to rt overnight (ca. 18h). The reaction was added slowly to rapidly stirred ice water resulting in a precipitate. The solid is filtered, washed with water and dried under vacuum to provide 7-bromo-1H-indole-3-carbonitrile as a red-brown solid (5.32 g, 94%). This material was used without further purification. ^{1}H NMR (500 MHz, DMSO-d6) δ 8.28 (s, 1H), 7.76 (s, 1H), 7.60 (d, J = 8.49 Hz, 1H), 7.37 (dd, J = 1.41, 8.49 Hz, 1H). ^{13}C NMR (126 MHz, DMSO-d6) δ 136.1, 135.6, 125.7, 124.6, 120.2, 115.9, 115.8, 115.6, 84.6. LCMS t_R=2.33 min, m/z 220.9, 222.8 $[M + H]^+$ 242.9, 244.9 $[M + Na]^+$.

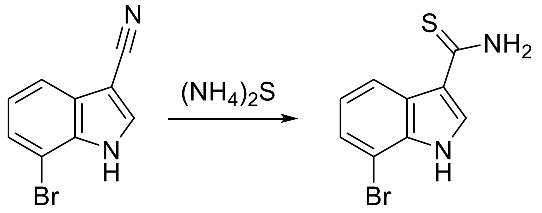

Synthesis of 7-bromo-1H-indole-3-carbothioamide. This procedure is a slight modification to that reported by Bagley and coworkers (2). To a 5 ml Biotage vial were charged 7-bromo-1H-indole-3-carbonitrile (398 mg, 1.80 mmol, 1.00 equiv.), 20 wt% aq. $(NH_4)_2S$ (3.1 ml, 9.0 mmol, 5.0 equiv.) and MeOH (0.6 mL). After sealing the vial, the suspension was irradiated in a Biotage Initiator microwave for 3h at 100°C. After cooling the reaction mixture, the precipitate was filtered, washed with water and dried under vacuum to obtain pale yellow crystals (438 mg, 95% yield) of 7-bromo-1H-indole-3-carbothioamide. This material was used in the next step without further purification. ^{1}H NMR (500 MHz, DMSO-d6) δ 11.84 (br. s., 1H), 9.03 (br. s., 1H), 8.90 (br. s., 1H), 8.59

(d, J = 8.65 Hz, 1H), 8.09 (s, 1H), 7.62 (d, J = 1.73 Hz, 1H), 7.27 (dd, J = 1.73, 8.65 Hz, 1H). ^{13}C NMR (126 MHz, DMSO-d6) δ 193.2, 137.7, 128.4, 125.2, 123.7, 123.6, 116.3, 114.8, 114.6. LCMS t_R=2.07 min. m/z 257.0, 254.9 $[M + H]^+$.

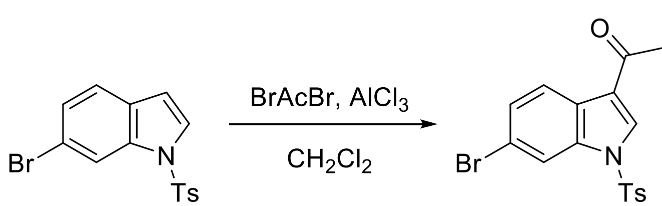

Synthesis of 2-bromo-1-(6-bromo-1-tosyl-1H-indol-3-yl)ethan-1-one. To a suspension of $AlCl_3$ (30.17 mmol) in CH_2Cl_2 (60 mL) was added bromoacetyl bromide (1440 uL, 16.5 mmol) at rt for 15 min. To this mixture was added a solution of 6-bromo-1-tosyl-1H-indole (7.31 mmol) in CH_2Cl_2 (10 mL) dropwise at 0 °C. The mixture was stirred at 0 °C for 1h and poured onto ice (60 g). The aqueous layer was extracted with CH_2Cl_2. The organic extracts were washed with saturated aq. $NaHCO_3$, brine and dried over anhydrous Na_2SO_4. After removal of the solvent, the crude product was recrystallized with $CHCl_3$/EtOH and column chromatographed with methylene chloride/hexane to afford 2-bromo-1-(6-bromo-1-tosyl-1H-indol-3-yl)ethan-1-one (2.32 g, 67% yield) as a white solid. ^1H NMR (500 MHz, CHLOROFORM-d) δ 8.30 (s, 1H), 8.16 (d, *J* = 8.49 Hz, 1H), 8.12 (d, *J* = 1.57 Hz, 1H), 7.82 - 7.87 (m, *J* = 8.49 Hz, 2H), 7.48 (dd, *J* = 1.65, 8.57 Hz, 1H), 7.30 - 7.37 (m, *J* = 8.17 Hz, 2H), 4.33 (s, 2H), 2.41 (s, 3H); ^{13}C NMR (125.7 MHz, CHLOROFORM-d) δ 186.8, 146.6, 135.4, 134.0, 133.0, 130.6, 128.6, 127.2, 126.4, 124.3, 120.0, 117.8, 116.3, 31.1, 21.8; LCMS m/z 471.6 $[M + H]^+$. Analytical and spectroscopic data were in agreement with those previously reported (3).

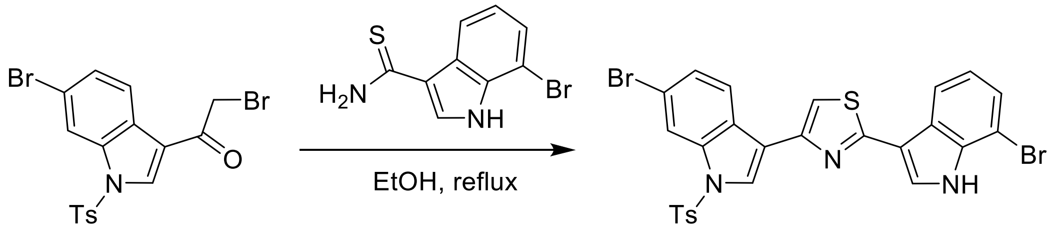

Synthesis of 4-(6-bromo-1-tosyl-1H-indol-3-yl)-2-(7-bromo-1H-indol-3-yl)thiazole. A suspension of 7-bromo-1H-indole-3-carbothioamide (815 mg, 3.19 mmol) and 2-bromo-1-(6-bromo-1-tosyl-1H-indol-3-yl)ethan-1-one (4) (1.58 g, 3.35 mmol) in EtOH (100 ml) was refluxed for 30 min. The mixture was cooled and the precipitates were filtered, washed with cold EtOH (20 mL x 2) and dried under vacuum to provide 4-(6-bromo-1-tosyl-1H-indol-3-yl)-2-(7-bromo-1H-indol-3-yl)thiazole (2.00 g, 99.9% yield) as a white solid. This material was used in the next step without further purification.

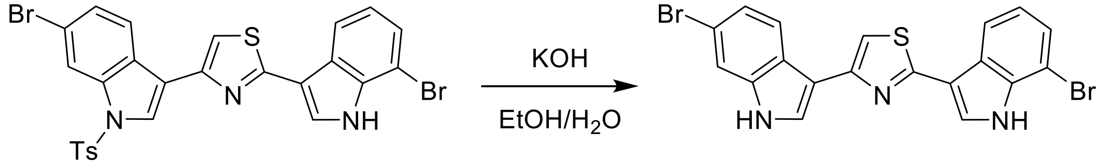

32-134D

Synthesis of 4-(6-bromo-1H-indol-3-yl)-2-(7-bromo-1H-indol-3-yl)thiazole (32-134D; FW = 473.2 g/mol). To a suspension of 4-(6-bromo-1-tosyl-1H-indol-3-yl)-2-(7-bromo-1H-indol-3-yl)thiazole (2.00 g, 3.19 mmol) in ethanol (40 ml) and water (40 ml), was added KOH (4 g, 5% W/V). The mixture was heated at reflux under nitrogen until complete (1 day). Water (40 mL) was added at RT to dilute the mixture. The reaction mixture was

filtered, washed with water (30 mL x 3) and the precipitate was dried. The precipitate was dissolved with EtOAc and EtOH and absorbed unto silica gel. Column chromatography (Hexane/(EtOAc + 10% EtOH)) provided 4-(6-bromo-1H-indol-3-yl)-2-(7-bromo-1H-indol-3-yl)thiazole (32-134D) (1.20 g, 79.4 % yield in two steps overall). ^{1}H NMR (500 MHz, DMSO-d6) δ 12.00 (br. s., 1H), 11.54 (br. s., 1H), 8.39 (d, J = 8.02 Hz, 1H), 8.18 (d, J = 8.65 Hz, 1H), 8.17 (s, 1H), 8.04 (s, 1H), 7.69 (s, 1H), 7.67 (s, 1H), 7.49 (d, J = 7.55 Hz, 1H), 7.30 (d, J = 8.65 Hz, 1H), 7.21 (t, J = 7.78 Hz, 1H); ^{13}C-NMR (125.7 MHz, DMSO-d_6) δ 161.8, 150.5, 138.0, 135.4, 127.9, 126.5, 126.3, 125.6, 124.2, 123.0, 122.7, 122.4, 120.6, 114.9, 114.8, 112.4, 111.9, 107.7, 105.3; LCMS m/z 471.6 $[M + H]^+$.

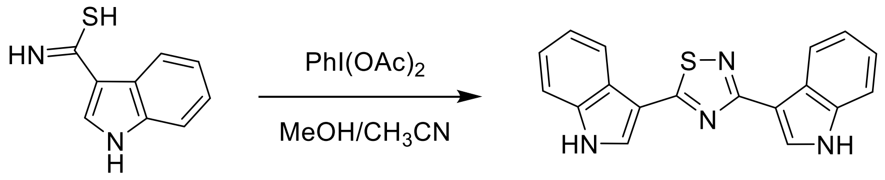

33-063

Synthesis of 3,5-di(1H-indol-3-yl)-1,2,4-thiadiazole (5, 6) *(33-063; FW = 316.4 g/mol)*. Iodobenzene diacetate (1.02 g, 2.41 mmol) was added to a stirred solution of indole-3-thiocarboxamide (416 mg, 2.36 mmol) in MeOH/CH_3CN (12 mL/12 mL) at 25 °C. The reaction was complete within 20 min. The reaction mixture was concentrated and filtered through celite washing with EtOAc. The EtOAc was concentrated *in vacuo* and the residue was purified by column chromatography on silica gel. 3,5-di(1H-indol-3-yl)-1,2,4-thiadiazole was obtained in a (275 mg, 74% yield). ^{1}H NMR (500 MHz, DMSO-d6) δ 12.13 (br. s., 1H), 11.72 (br. s., 1H), 8.47 (m, 1H), 8.43 (s, 1H), 8.27 (m, 2H), 7.57 (m, 1H), 7.53 (m, 1H), 7.32 (m, 2H), 7.23 (m, 2H)); ^{13}C NMR (125.7 MHz, DMSO-d_6) δ 180.7, 170.2, 137.2, 137.1, 129.8, 129.2, 125.6, 124.7, 123.4, 122.6, 122.0, 121.0, 120.7, 113.1, 112.5, 110.7, 108.1; LCMS m/z 317.0 $[M + H]^+$.

Bioanalytical method for mouse pharmacokinetics. Plasma (25 µL) was added to a borosilicate glass test tube and mixed with 150 µL of acetonitrile containing the internal standard (1 ng/mL of EXP-3179). For blank samples, 150 µL of acetonitrile was added without internal standard. Samples were vortex-mixed and centrifuged (1200 ×g for 5 minutes at ambient temperature) and transferred to an autosampler vial. Then 2 µL was injected onto the liquid chromatography system using a temperature-controlled autosampling device operating at approximately 10°C. Chromatographic analysis was performed using a Waters Acquity ™ Ultra Performance LC. Separation of the analyte from potentially interfering material was achieved at ambient temperature using Halo C18 column (50 x 2.1 mm i.d.) with a 2.7-µm particle size. The mobile phase used for the chromatographic separation was composed of 0.1% (v/v) formic acid in water (mobile phase A) and 0.1% (v/v) formic acid in acetonitrile (mobile phase B) with a flow rate of 0.4 mL/minute. The initial mobile phase composition was 60% mobile phase A and 40% mobile phase B. From 0.5 to 2.0 minutes, mobile phase B was increased linearly from 40% to 100% and maintained until 3.0 minutes. From 3.0 to 3.1 min, the gradient decreased to 40% mobile phase B and the conditions were maintained until 5 minutes to re-equilibrate the column for the next injection. The column effluent was monitored using an AB Sciex Triple Quadrupole 5500 mass spectrometer. The instrument was equipped with an electrospray interface, operated in a positive mode and controlled by the Analyst

v1.7 software. The settings were as follows: curtain gas 20 psi, medium collision gas, ion spray voltage 5500 V, probe temperature 450°C, ion source gas one 30 psi, ion source gas two 40 psi, and entrance potential 10. The collision cell exit potentials were 14.0 and 6.0 for 32-134D and the internal standard, respectively. The declustering potential was 141 and 80 for 32-134D and the internal standard, respectively. The collision energies were 43 and 25 for 32-134D and internal standard, respectively. MRM m/z transitions were the following: 474.6 → 393.8 and 421.0 → 207.1 for 32-134D and the internal standard, respectively. Dwell time was 150 milliseconds. The calibration curve for 32-134D was constructed from the peak area ratio of the analyte to the peak area of its internal standard (EXP-3179) using the least-squares quadratic regression analysis with $1/x^2$ weight over the range of 0.01-2.11 µM with dilutions of up to 1:10 (v/v).

References

1. Vorbruggen H, and Krolikiewicz K. The Introduction of Nitrile-Groups into Heterocycles and Conversion of Carboxylic Groups into their Corresponding Nitriles with Chlorosulfonylisocyanate and Triethylamine. *Tetrahedron*. 1994:50(22):6549-6558.
2. Bagley MC, Chapaneri K, Glover C, Merritt EA. Simple Microwave-Assisted Method for the Synthesis of Primary Thioamides from Nitriles *Synlett*. 2004;(14):2615–2617.
3. Guo J, et al. Optimization, Structure-Activity Relationship, and Mode of Action of Nortopsentin Analogues Containing Thiazole and Oxazole Moieties. *J. Agric. Food Chem.* 2019;67(36):10018–10031.
4. Jiang B, and Gu XH. Syntheses and cytotoxicity evaluation of bis(indolyl)thiazole, bis(indolyl)pyrazinone and bis(indolyl)pyrazine: Analogues of cytotoxic marine bis(indole) alkaloid. *Bioorganic Med. Chem.* 2000;8(2):363–371.
5. Pedras MSC, Hossain S, and Snitynsky RB. Detoxification of cruciferous phytoalexins in *Botrytis cinerea*: Spontaneous dimerization of a camalexin metabolite. *Phytochemistry*. 2011;72(2-3):199–206.
6. Kumar D, Kumar NM, Chang KH, Gupta R, Shah K. Synthesis and in-vitro anticancer activity of 3,5-bis(indolyl)-1,2,4-thiadiazoles. *Bioorganic Med. Chem. Lett.* 2011;21(19):5897–5900.
